# Mechanism of Immune Evasion in Mosquito-Borne Diseases

**DOI:** 10.3390/pathogens12050635

**Published:** 2023-04-23

**Authors:** Swagato Bhattacharjee, Debanjan Ghosh, Rounak Saha, Rima Sarkar, Saurav Kumar, Manoj Khokhar, Rajan Kumar Pandey

**Affiliations:** 1 DBT Rajiv Gandhi Centre for Biotechnology, Thiruvananthapuram 695014, India; 2 Department of Biotechnology, Pondicherry University, Puducherry 605014, India; 3 Department of Biochemistry and Molecular Biology, Pondicherry University, Puducherry 605014, India; 4 Department of Biochemistry, AIIMS, Jodhpur 342005, India; 5 Department of Medical Biochemistry and Biophysics, Karolinska Institute, 171 77 Solna, Sweden

**Keywords:** mosquito-borne diseases, malaria, dengue, Zika, chikungunya, West Nile fever, Rift Valley fever, virus

## Abstract

In recent decades, mosquito-borne illnesses have emerged as a major health burden in many tropical regions. These diseases, such as malaria, dengue fever, chikungunya, yellow fever, Zika virus infection, Rift Valley fever, Japanese encephalitis, and West Nile virus infection, are transmitted through the bite of infected mosquitoes. These pathogens have been shown to interfere with the host’s immune system through adaptive and innate immune mechanisms, as well as the human circulatory system. Crucial immune checkpoints such as antigen presentation, T cell activation, differentiation, and proinflammatory response play a vital role in the host cell’s response to pathogenic infection. Furthermore, these immune evasions have the potential to stimulate the human immune system, resulting in other associated non-communicable diseases. This review aims to advance our understanding of mosquito-borne diseases and the immune evasion mechanisms by associated pathogens. Moreover, it highlights the adverse outcomes of mosquito-borne disease.

## 1. Introduction

**Figure 1:**
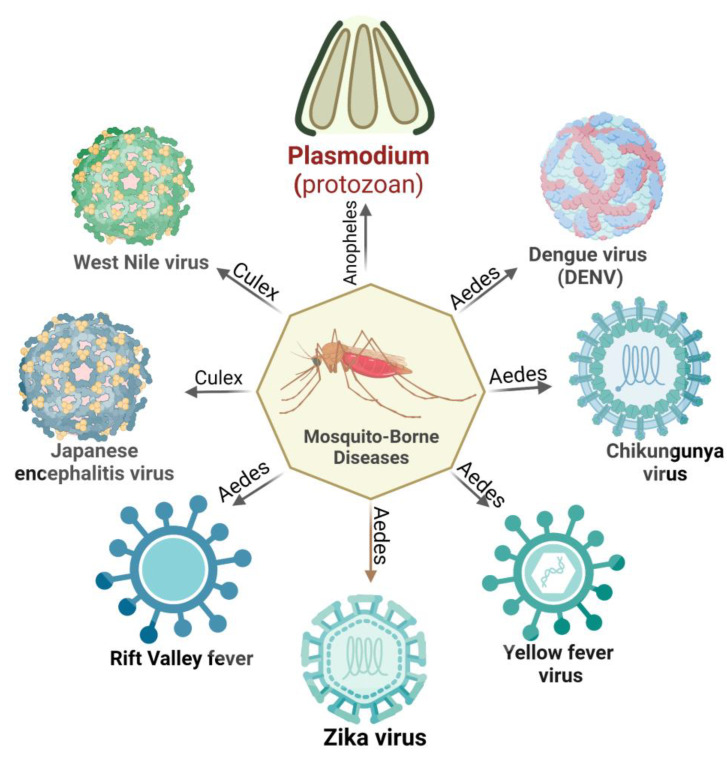
Different pathogens involved in mosquito-borne diseases include Plasmodium, dengue virus, chikungunya virus, yellow fever virus, Zika virus, Rift Valley fever virus, Japanese encephalitis virus, and West Nile virus.

Vector-borne diseases are a major public health concern worldwide, accounting for a substantial proportion of the global disease burden. As per a World Health Organization (WHO) report, nearly 700,000 deaths are caused annually by vector-borne contagious diseases [1]. Mosquitoes are a potential vector for disease transmission and can disproportionately impact impoverished communities, particularly children. Regardless of acquiring centuries of control strategies, mosquito-borne diseases are burgeoning. These conditions are responsible for enormous mortality and morbidity worldwide [2] (Figure 1).

Among mosquito-borne diseases, malaria is a potentially fatal disease caused by *Plasmodium* parasites and transmitted by hitherto infected female anopheles mosquito bites. When the vector mosquito feeds on an infected person’s blood, *Plasmodium* parasites get ingested along with the blood meal. Subsequent bites to a healthy person may transmit the parasite, which leads to malaria. Its symptoms begin with the first liver schizont rupture and merozoite release in the peripheral circulation [3]. Five species of the malarial parasite have been reported, *Plasmodium falciparum*, *P. vivax, P. ovale, P. malariae*, and *P. knowlesi*, leading to clinical symptoms in humans. *P. falciparum* is the most lethal species associated with the majority of malaria-related deaths worldwide. Apart from its ability to impair neuro-cognitive functions, this species is known to develop resistance to anti-malarial drugs. *P. vivax,* the most widely distributed species, is responsible for a significant proportion of malaria cases worldwide. Other *Plasmodium* species, namely *P. ovale,* differ in the latency period and resemble *P. vivax* clinically [3]. *P. malariae* is a relatively rare species but has distinct clinical outcomes. Compared to other malaria types, the number of merozoites produced with each schizont rupture is lower; thus, the parasitemias are lower in these patients [4]. Because of the longer parasite life cycle, patients experience fever every 72 h during an infection. *P. knowlesi,* despite its limited distribution, shows a higher severity rate than other common strains [5].

Mosquitoes can also harbor viral pathogens and cycle them between the human population. Among mosquito-borne viral infections, dengue is the most common disease caused by dengue virus 1–4 (DENV 1–4). DENVs are most commonly spread through the bite of an infected female *Aedes* sp. mosquito. A person infected with a particular dengue virus serotype can sometimes induce short-term cross-reactivity with other serotypes as well. Although the majority of dengue virus infections are asymptomatic or only cause mild disease, severe disease can occur and is characterized by plasma leakage, a pathophysiologic process in which the protein-rich fluid component of blood leaks into the surrounding tissue, resulting in extravascular fluid accumulation and shock, coagulopathy, and end-organ impairment [6].

Chikungunya virus (CHIKV), a Togaviridae with a single-stranded, positive-sense RNA genome, is transmitted mainly by the mosquito vector *Aedes aegypti* and, to some extent, *A. albopictus,* mainly in regions of Paraguay, Bolivia, Argentina, and Thailand [7]. Patients infected with CHIKV generally encounter intense asthenia, myalgia, headache, and arthralgia. Unlike other acute infections, CHIKV infection is dedicated to attacking the skeletal muscles, joints, and myotendinous insertions [8].

Yellow fever virus (YFV), a flavivirus causing yellow fever, has been considered one of the deadliest infectious diseases. Endemic to the tropics and sub-tropics, this virus is transmitted via *Haemogogus janthinomys*, *H. leucocelaenus, Sabethe*, and *Aedes* mosquitoes. Naturally, YFV circulated between mosquitoes and non-human primates in a sylvatic cycle. Following urbanization, YFV has entered the urban cycle, infecting humans and resulting in human-to-human circulation [9]. *A. africanus* maintains a sylvatic cycle in the rainforest, whereas *A. bromelia* was found to serve as a vector mediating urban cycles. The subsequent return of sick individuals to non-endemic, more densely populated places can set off an urban cycle perpetuated by *A. aegypti* mosquitoes [10]. Individuals typically experience an incubation period of 3 to 6 days after being bitten by an infected mosquito, followed by flu-like symptoms before a remission period of 1 to 2 days. Following remission, some patients (20–60%) progress to a more toxic phase of the disease, characterized by hemorrhagic fever, jaundice, thrombocytopenia, and liver and renal failure [11].

Zika virus (ZIKV) is an arbovirus that usually causes asymptomatic infections in the human host, but several neurological impairments have been reported in several cases. ZIKV is transmitted in the human population mainly through bites of anthropophilic mosquitoes such as *A. aegypti*, *A. albopictus*, and *A. hensilii.* Its urban cycle uses humans as reservoirs and continuously multiplies in them. Apart from this cycle, a sylvatic transmission cycle operates between non-human primates and arboreal canopy-dwelling mosquitoes. ZIKV can persist in mosquito eggs, leading to transovarial transmission, where the virus infects ovarian tissues, and transegg transmission, where the virus infects its host during fertilization [12]. Besides the direct human-to-human transmission, ZIKV has also been found to travel to fetuses from infected mothers *(*in utero).

Other mosquito-transmitted viruses, including the Rift Valley fever virus (RVFV) and Japanese encephalitis virus (JEV), follow the epizootic transmission cycle, where the virus amplifies in domesticated animals before infecting humans. Rift Valley fever is an emerging zoonotic viral disease caused by the RVFV of the Bunyaviridae family. Cattle, sheep, goats, and camels are especially vulnerable to RVF and serve as viral amplification hotspots. Infection of domestic animals is initiated by female *Aedes* mosquitoes (the primary mosquito vectors) with a disseminated salivary gland with an RVFV infection during probing or blood-feeding. After the primary infection, *Culex* and *Mansonia* sp. can channel RVFV between viremic domestic animals and humans [13]. JEV, the causative agent of human encephalitis, is primarily transmitted by *Culex tritaeniorhynchus* and *C. annulirostris*. *C. pipiens* and *A. japonicus* could be considered potentially important vectors in the case of JEV introduction in new regions [14].

West Nile virus (WNV), another emerging neurotropic flavivirus, is predominantly found to cycle between mosquitoes and birds. *Culex pipens* and *C. quinqifasciatus* serve as WNV vectors in major parts of Asia, Africa, and America, whereas *C. australicus* and *C. globcoxitus* have been found to be predominant in Australia. While feeding on infected viraemic birds, female Culex spp. mosquitoes pick up WNV. The virus replicates in the mosquito’s midgut epithelial cells and spreads to the salivary glands and other organs via hemolymph [15]. The pathogenesis of WNV includes an initial infectivity period followed by viral amplification and, finally, neuro-invasion in the central nervous system [16].

From the mosquito perspective, *A. aegypti* is distributed worldwide and found in most tropical countries. *A. albopictus*, on the other hand, is an opportunistic daytime and outdoor feeder that prefers humans and can be found feeding and resting indoors. Vertical transmission (VT) of viral pathogens is common in mosquito hosts. Similarly, viruses can also spread among mosquito populations through venereal transmission, which involves viral transfer during mosquito mating. Males cannot contract the virus through a blood meal, but they can contract it through venereal transmission from an infected female partner. Transmission by different species depends on some specific alterations in their non-structural proteins. *Aedes aegypti* was found to be more prone to ZIKV infection owing to a mutation in its non-structural protein 1 (NS1), whereas *A. triseriatus* and *A. taeniorhynchus* were not susceptible [17,18]. Other *Aedes* species, such as *A. furcifer,* were found to transmit DENV to humans, whereas *Aedes luteocephalus* transmit DENV and YFV in non-human primates [19]. In this context, several cases of co-infection were reported where patients were positive for both CHIKV and DENV [20]. In the genus *Culex*, *C. quinquefasciatus*, the most abundant species in tropical Africa, serves as a vector for the transmission of CHIKV and Japanese encephalitis virus (JEV). Meanwhile, *C. antennatus* is speculated to play an essential role as an epizootic vector of WNV [21]. The *Anopheles* genera support the transmission of *Plasmodium* sp. and certain nematodes but do not support arboviral transmission [22]. This review explains the complex mechanisms utilized by mosquito-borne pathogens to evade host immune systems. Malaria, Dengue fever, chikungunya, and other illnesses present substantial health challenges in tropical regions. Additionally, this review investigates the effects of these evasion strategies on disease progression and other outcomes. Through analysis of current scientific discoveries, we aim to enhance the understanding of the intricate interplay between these pathogens and the host’s immune responses, ultimately contributing to developing effective strategies to tackle these diseases and improve global health.

## 2. Immune Evasion in Mosquitoes

Mosquito midgut tissues are the first to be infected [23] and therefore have been the primary area of focus for studying the immune responses of the vector. Researchers aim to identify the molecules and pathways involved in the infection process of mosquito-borne pathogens. The major innate immune pathways involved in vector immune responses are the toll pathway, IMD pathway, JAK-STAT pathway, RNAi pathway (mi-RNA and pi-RNA pathways), and the interferon-mediated antiviral response. Mosquito-borne pathogens can shut down all these defense mechanisms by downregulating the responsible genes for these pathways [24,25]. According to a transcriptome study, the toll pathway plays a role in restricting the mosquito’s capacity for infection. Toll, Rel1A, and Spätzle (Spz) are the genes responsible for Toll activation, whereas cactus is the negative regulator.

In the mosquito, the anti-plasmodial response in *Anopheles* is enacted by the circulating thioester-containing protein 1 (TEP 1) responsible for humoral immunity at the ookinete stage. Apart from this, Enterobacter, Pseudomonas, Asaia, and Panteoa induce the secretion of antimicrobial peptides (AMPs) against the invading *Plasmodium* [26]. The innate immune system of *Anopheles* controls pathogen invasion by regulating three signaling cascades: the immune deficiency, the Toll, and the JAK-STAT pathways [27]. Immune evasion by *Plasmodium* includes the activation of the Pfs47 gene, which allows the parasite to inhibit several caspases responsible for the Jun-N-terminal-kinase-mediated activation of mid-gut apoptosis [28]. Furthermore, Pfs47 inhibits midgut nitration responses, which are required to activate the complement-like system [29].

Arboviruses can inhibit the antibacterial and antiparasitic activity of the IMD pathway in *Aedes aegypti*, leading to enhanced viral replication [30]. Functional studies have revealed that genes involved in the JAK-STAT pathway are essential to the vector’s immune system. This pathway is upregulated as an early response to arbovirus infection [31]. However, DENV and other mosquito-borne pathogens have evolved mechanisms to downregulate the JAK-STAT immune response [24]. Interfering RNA is one of the most successful antiviral defense mechanisms in mosquitoes [32]. Different viral proteins/factors can inhibit the vector’s RNAi processing pathways to prevent the degradation of their genomic material. The cytokine-like element named Vago establishes coordination between an IFN-like antiviral immunity pathway and the canonical innate immunity pathway (JAK-STAT) in culex mosquitoes [33].

## 3. Immune Evasion in the Vertebrate Host

### 3.1. Immune Response at the Skin Barrier

Immunity at the skin level is a crucial first line of defense against pathogen invasion. This is especially true for vector-borne diseases such as malaria, dengue, chikungunya, yellow fever, Zika virus infection, Rift Valley fever, Japanese encephalitis, and West Nile virus infection. Post-pathogen entry via mosquito bite into host skin, the immune response at the skin level plays a vital role in preventing the establishment of the infection [34]. This response involves activating local immune cells, such as dendritic cells, macrophages, and natural killer cells, which recognize and eliminate pathogens. The skin also contains antimicrobial peptides and proteins that can kill or neutralize invading pathogens [34]. The skin’s immune response is complex and can be modulated by various factors, including age, sex, and previous exposure to the pathogen. Understanding the skin’s immune response to these vector-borne pathogens is critical for developing effective preventive and therapeutic strategies. In the case of malaria, the sporozoites are blocked at the dermis and gradually removed by phagocytic cells. SPECT-1 (sporozoite microneme protein essential for cell traversal) and SPECT-2 (perforin-like protein 1) proteins are used by these sporozoites to avoid dermal blockade and phagocytic ingestion by immune cells [35]. Numerous mosquito-borne viruses, such as DENV and WNV, actively replicate in migratory Langerhans cells (LCs), thereby indicating that this ability might be a desirable method inside vertebrate hosts [36]. Different receptors have been found or suggested as entry points for DENV, which enters the cell through receptor-mediated endocytosis. It has been demonstrated that DENV can be bound in vitro by heparin sulfate, proteoglycan, and glycosaminoglycans, which are frequently expressed on different mammalian cell types [37].

### 3.2. Immune Evasion Strategies by Plasmodium Parasites

**Figure 2:**
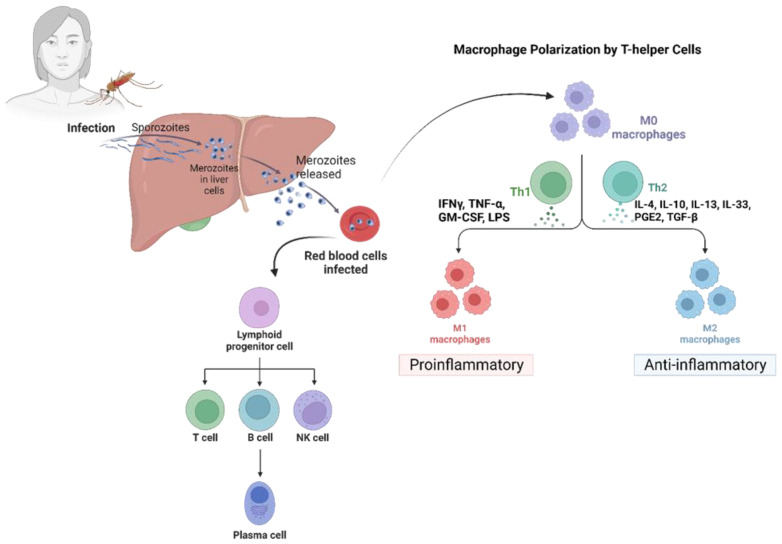
*Plasmodium* and infected red blood cells activate dendritic cells through parathyroid hormone (PTH)/PTH-related peptide type 1 receptor (PPR) and are phagocytosed, and their antigens are presented to T cells. PRR signaling leads to the secretion of cytokines that initiate inflammation via Th1 and Th2 cell differentiation and macrophage polarization. Macrophages are responsible for the regulation of inflammation during the infection phase. T cells help with B cell differentiation and antibody secretion and secrete IFN-γ, which activates macrophages. IFN-γ-activated macrophages engulf opsonized cells.

Malaria is a major public health concern and is most prevalent in tropical Africa [38]. The primary survival strategy adopted by *Plasmodium* species inside the human host is immune evasion. This involves a common mechanism wherein parasitic proteins interact with specific receptors on the erythrocyte’s surface. This mechanism is shared by all *Plasmodium* species [39]. Malaria symptoms and clinical manifestations typically become apparent during the asexual reproduction of the parasite within the red blood cells (RBCs) of the vertebrate host [40]. The parasite has adapted to modify RBCs’ morphology and surface features to evade the host’s immune system. Other mechanisms to evade the host’s immune system involve the suppression of Kupffer cells and dendritic cells (DCs) [41] (Figure 2).

Among the five *Plasmodium* species, *P. falciparum* is the most severe, causing high morbidity and mortality, and is found predominantly in sub-Saharan Africa, South-East Asia, and Eastern Mediterranean countries. *P. vivax* is the second most widespread species, causing moderate illness, and is distributed throughout Ethiopia, Sudan, Guatemala, Brazil, Colombia, Indonesia, Myanmar, India, Pakistan, Papua New Guinea, and Afghanistan. *P. ovale*, a less severe species, causes low to moderate malaria cases and is mainly endemic to tropical Western Africa. *P. malariae*, which causes mild malaria, is distributed across sub-Saharan Africa and the southwest Pacific. Lastly, *P. knowlesi*, a zoonotic species, causes moderate- to high-severity malaria and is primarily found in Southeast Asian regions of Malaysian Borneo, extending to peninsular Malaysia [42].

There is a significant difference in disease severity between *P. falciparum* and *P. vivax*. The former causes severe malaria, characterized by a high parasitemia level and the sequestration of infected red blood cells (RBCs) in the microvasculature of different organs, including the brain, lungs, and placenta [43]. In contrast, the latter has a lower parasitemia level and does not sequester in organs, leading to a less severe disease. However, *P. vivax* uniquely forms hypnozoites in the liver, which is a dormant stage that causes relapses [44,45].

Studies suggest that the immune response to *P. falciparum* and *P. vivax* infections differs. *P. falciparum* infections result in a strong proinflammatory response, essential for controlling the infection but also causing tissue damage and contributing to severe malaria pathogenesis. *P. vivax* infections, on the other hand, elicit a less intense immune response, which may be related to the low parasitemia levels observed during infections [46]. Additionally, it can modulate the host’s immune response to promote its survival, for example, by reducing proinflammatory cytokine levels [47]. In an earlier study, blood was taken for inspection to compare the immune response against *P. vivax*- and *P. falciparum*-infected patients. This whole blood sample was taken before and after infection once the count exceeded 10,000 parasites/mL. CD38 is a surface glycoprotein marker that facilitates signal transduction and cell adhesion and regulates Ca2^+^ levels that are upregulated on lymphocytes after activation [48]. When comparing the frequencies of CD38^+^ T cells, it was noted that *P. falciparum* infection was associated with higher numbers of CD38^+^ CD4^+^ T cells, whereas *P. vivax* infection was associated with higher numbers of CD38^+^ CD8^+^ T cells. However, no discernible differences in the frequency of CD38^+^ B cells were observed, irrespective of the presence of *P. falciparum* or *P. vivax* [49]. Little is known regarding the immune response to the dormant hypnozoite stage of *P. vivax*. However, recent studies suggest the immune response to hypnozoites is different from that of the blood-stage parasite, and the host’s immune system may not be able to recognize or eliminate dormant hypnozoites effectively [50]. *P. falciparum* and *P. vivax* differ significantly in their ability to cause disease, sequestrate in organs, and form dormant stages. These differences also affect the immune response to infections [51] (Table 1).

#### 3.2.1. Immune Evasion Strategies at the Liver Stage

Malaria’s pre-erythrocytic and blood stages are initiated when a female Anopheles mosquito carrying the malaria parasite feeds on human blood [40,55]. Post mosquito bites, sporozoites are discharged into the victim’s dermis and enter into the bloodstream, where they must surpass Kupffer cells (KC) and endothelial cells (EC) to invade the hepatocytes and start an infection [55,56]. Sporozoites can also pass through the gaps between EC and KC by modulating cytokinin activity, upregulating anti-inflammatory Th2 cytokines, and downregulating inflammatory Th1 cytokines [57,58]. Additionally, sporozoite-produced circumsporozoite protein (CSP) can interact with KC surface proteins, namely LRP-1 (low-density lipoprotein receptor-related protein) and proteoglycan, and inhibit ROS production by producing intracellular cAMP/EPAC [58]. Notably, the sporozoites induce KC apoptosis, while the CSP reduces KC-specific expression of MHC-1, decreasing its antigen presentation capability [59]. Thus, the sporozoites can effectively minimize KC functions and make their entry into liver hepatocytes [39].

#### 3.2.2. Immune Evasion in the Hepatocyte Stage

The sporozoites successfully enter the hepatocytes after infiltrating the sinusoidal layer and downregulate the mTOR pathway, thus affecting the proteins needed for cell division, proliferation, and autophagy. The sporozoites always travel through several hepatocytes until they reach a final hepatocyte, where they develop into a merozoite [60]. Hepatocyte growth factor (HGF) is secreted by the transmigrated hepatocytes, which bind the c-MET receptor and makes the hepatocyte vulnerable to infection by rearranging the host cell’s actin cytoskeleton [61]. By upregulating the PI-3 kinase/MAPK and ATK pathway, this interaction confers resistance to apoptosis in the hepatocyte [62]. Sporozoite-released CSP binds to high-sulfate HSPGs (heparan sulfate proteoglycans) on hepatocytes and undergoes cleavage, exposing the TSR (thrombospondin repeat) domain. The binding of the TSR domain to HSPG on hepatocytes facilitates the invasion of the hepatocytes by sporozoites [63,64]. The sporozoite is encased in a parasitophorous vacuole to protect them from host lysosomal degradation. The cleaved CSP makes its way into the cytoplasm of the hepatocytes using its PEXEL domain [65]. Cleaved CSP inhibits host cell protein synthesis, benefiting sporozoite survival [66]. It also suppresses the NF-κB signaling pathway [65] and significantly lowers p53 expression in the infected hepatocytes, allowing parasite survival [67].

#### 3.2.3. Immune Evasion Strategies at the Pre-Erythrocytic Stage

The invasive form of the parasite, the merozoites [68], leads to the asymptomatic phase of the disease, referred to as the pre-erythrocytic stage. Merozoites save themselves from resident KC and DC by covering themselves in merosomes, which are derived from the host hepatocytes [69]. Following maturity, the merosomes (vesicles containing merozoites) bud off the hepatocyte and are released into the bloodstream [69]. This initiates the erythrocytic stage of the infection. Subsequently, initiating the erythrocytic stage of infection can take several days after the exit of merosomes from the liver [39].

#### 3.2.4. Immune Evasion Strategies at the Erythrocytic Stage

Following the release of merozoites into the bloodstream, they invade the host RBCs and begin their maturation phase within the RBCs. The merozoite matures into a trophozoite within a membrane-bound parasitophorous vacuole inside the host RBC [41], which further undergoes schizogony to produce the multinucleated schizont stage containing daughter merozoites [70]. These infected RBCs (iRBCs) release their merozoites into the bloodstream upon maturation, which continues to infect new RBCs. This mechanism leads to the development of the malarial parasite’s asexual stage inside of host RBCs [41].

#### 3.2.5. Antigenic Variation on the Surface of iRBCs

The iRBCs evade the host’s immune response by expressing various surface antigens encoded by multigene families known as variant surface antigens (VSA) [71,72,73,74,75]. Three VSAs have been identified in *P. falciparum*, namely *P. falciparum* erythrocyte membrane protein 1 (PfEMP1) coded by the var genes, repetitive interspersed repeats (RIFIN) coded by the rif genes, and sub-telomeric variant open reading frame (STEVOR) coded by the stevor genes [76].

There are approximately 60 var genes that express PfEMP1 [77] on RBC surfaces [78]. Approximately 150 rif genes per genome are clustered at the sub-telomeric region of the chromosome [77,79]. RIFINs are classified into two sub-families, namely A-type RIFIN and B-type RIFIN. The former are trafficked to the iRBC membrane with the help of the PEXEL motif, while the latter is present inside the parasite [80,81]. Lately, RIFINS were also found in merozoites with A-type RIFIN, localizing the apical tip and B-type RIFIN present in the cytoplasm of merozoites [82]. RIFINS have evolved host immune evasion mechanisms, such as rosetting, which will be discussed in the next section. The STEVOR protein family is similar to RIFIN and comprises 28 copies of the stevor gene in the *P. falciparum 3D7* reference strain [77]. STEVOR also contains a PEXEL motif that targets the proteins involved in export to the surface of the iRBC membrane [83].

#### 3.2.6. Cytoadherence and Sequestration as an Immune Evasion Strategy

The spleen clears out abnormal and old RBCs and kills bloodborne pathogens, which places the circulating iRBCs in grave danger [41]. The *Plasmodium*-infected RBC bind to human endothelial cells and then evade the spleen via a process known as cytoadherence. This causes the iRBCs to sequester in the microvasculature of various organs [41]. The var gene-encoded PfEMP1 mediates the sequestration of iRBCs [84]. The antigenic variability and cytoadhesive properties of iRBCs are contributed by the Duffy binding ligand (DBLs) and cysteine-rich interdomain region (CIDRs) domains of the PfEMP1 proteins [85,86,87]. The acid terminal segment (ATS) domain assists in projecting PfEMP1 onto the surface of RBCs [88]. PfEMP1 also binds to numerous host cell receptors, such as intercellular adhesion molecule 1 (ICAM1), CD36, chondroitin sulfate A (CSA), endothelial protein C, heparin sulfate, IgM, α-2 macroglobulin, thrombospondin, and complement receptor 1 (CR1), and promoting the process of cytoadherence and sequestration [89,90,91,92,93,94,95,96]. Sequestration and cytoadherence allow the iRBCs to adhere to endothelial cells, which protects them from being cleared by the spleen.

#### 3.2.7. Rosetting as an Immune Evasion Strategy

Rosetting is the process where iRBCs adhere to healthy RBCs and form a rosette-like structure. It helps the iRBCs to elude the host’s immune system and shields the newly released merozoites against host-invasion-inhibitory antibodies [97]. It also provides a suitable microenvironment for the emerging merozoites to invade the healthy RBCs [98]. Recent literature has demonstrated that STEVOR, RIFIN, and PfEMP1 can induce rosetting by binding to glycophorin C and blood group A antigen, respectively [99]. This shows that rosette formation is mediated by three different strategies the parasite has evolved, indicating a crucial role in parasite survival.

Different variants of PfEMP1 bind to different RBC receptors, such as CR1, heparin sulfate, and α2 macroglobulin, and mediate the process of rosetting [94,100,101,102,103]. It has been shown that PfEMP1 binds to both α2 macroglobulin and IgM [104]. The binding of IgM increases the avidity of iRBCs, thus mediating rosetting [105]. Recent studies also show that cytoadhesion and rosetting can be mediated simultaneously by a single PfEMP1 molecule from the *P. falciparum* rosetting strain [96].

A study by Goel et al. demonstrated that one member of the A-type RIFIN family could form large rosettes by adhering to the blood group A antigen on RBCs [106]. RIFINs can also form smaller rosettes by binding to the glycophorin A receptor on the surface of O blood group RBCs [106]. In addition, RIFIN-mediated rosettes seem to shield PfEMP1 from antibody identification, suggesting their function as protective antigens and in immune evasion [106,107]. Recent research has demonstrated that STEVOR can independently promote rosetting without PfEMP1 by binding to glycophorin C on healthy RBCs [108,109]. The study conducted by Niang et al. also proved that merozoites are protected from invasion-inhibitory antibodies with the aid of STEVOR-mediated rosettes [99,108]. Rosetting suggests that *Plasmodium* species has used this mechanism for its survival and efficient transmission in the host.

#### 3.2.8. Malaria and Its Association with Other Disease Outcomes

*Cancer*: Although cancer and malaria have been historically studied separately, recent evidence suggests the importance of investigating their potential biological interactions in light of their evolutionary history and epidemiology. According to epidemiological data, there appears to be an inverse relationship between cancer and several mosquito-borne diseases, whereby cancer cases rise as the chances of contracting mosquito-borne infections decrease [110]. Parasites may alter the balance between immunosuppression and immunity against a tumor by modifying the availability and presentation of cross-reactive antigens, influencing the induction of pre-existing immunity, and modifying the components of the tumor microenvironment [111]. Malaria pathogenesis may selectively induce certain immunological events, such as the production of inflammatory cytokines, that can potentially lead to cancer [112]. p53, the master regulator of oncogenesis, is well known for its role in cell cycle regulation and apoptosis. Kaushansky et al. observed a reduction in p53 levels during malarial parasite infection and demonstrated that this decrease in p53 levels led to an increase in hepatic infection in mice as their model organism. Conversely, they found that boosting p53 levels significantly reduced hepatocyte infection [67]. The decrease in p53 activity observed during malarial parasite infection could potentially contribute to developing hepatic cancer. In addition, *P. vivax*, in its sporozoite stage, utilizes the Duffy antigen receptor for chemokines (DARC) receptor to invade red blood cells. Interestingly, the same receptor has been found to sequester chemokines that are essential for tumor metastasis and angiogenesis in a breast cancer model [113]. In addition, DARC has been found to interact with the tumor suppressor protein KAI1 (CD82), which plays a crucial role in inhibiting tumor cell proliferation and promoting senescence. This interaction was supported by direct evidence obtained through DARC knockout mice, which demonstrated that the metastasis-arresting property of CD82 was severely compromised [114]. Malarial parasites attach to the placenta using variant surface antigens during pregnancy. Interestingly, these same antigens have been found to interact with chondroitin sulfate A (CSA), a glycosaminoglycan that enhances aggressiveness and metastasis [115,116]. Epidemiologically, malarial infection has been associated with Burkitt’s lymphoma (eBL) [117]. Post-infection, Robbiani et al. reported the rapid clonal proliferation of B cells, which can lead to genomic instability and the alteration of B cells to non-Hodgkin’s lymphoma through the action of an activation-induced deaminase, an enzyme responsible for DNA mutations and double-stranded breaks [118]. In a rodent model displaying immunity against the malarial parasite, Burkitt’s lymphoma was observed after repeated *Plasmodium* infections, suggesting that the immune response against malaria could act as a switch leading to eBL in the presence of Epstein–Barr virus (EBV) [119].

*Cardiovascular diseases*: Evidence suggests *Plasmodium* infections, specifically those caused by *P. falciparum*, may increase the risk of cardiovascular disease and heart attacks. This is thought to occur through the chronic inflammation and endothelial dysfunction that can result from repeated or chronic malaria infections [120,121]. Brainin et al. conducted a cohort study in Denmark and found that individuals with *Plasmodium* infections had an increased risk of heart attack [122]. Cardiovascular complications in malaria are mainly attributed to the altered cytoadhesive properties of *P. falciparum*-infected erythrocytes. These parasites use platelet bridges to connect infected red blood cells (RBCs) and ultimately form aggregates through autoagglutination, leading to microvascular obstruction, ischemia, and tissue damage. This mechanism of cytoadhesion and sequestration of infected RBCs in the microvasculature has been implicated in the pathogenesis of severe malaria syndromes, including cerebral malaria and acute respiratory distress syndrome (ARDS), which are characterized by widespread endothelial dysfunction and multiorgan failure [122,123]. Furthermore, these agglutinated aggregates accumulate in myocardial capillaries, obstructing blood flow and contributing to the progression of cardiovascular disease. Additionally, Wennicke et al. found that plasmodial glycosylphosphatidylinositol (GPI), which is involved in the pathogenesis, can lead to apoptosis in cardiomyocytes [124]. Plasmodial GPI has been shown to induce the production of pro-inflammatory cytokines such as TNF-α and IL-6, which can cause excessive nitric oxide (NO) production. This NO overproduction can lead to apoptosis and inflammatory cardiomyopathy, characterized by contractile dysfunction and myocardial energy depletion. Therefore, it is suggested that the pathogenesis of malaria-related cardiovascular complications involves the dysregulation of cytokine and NO production through GPI-mediated mechanisms [125,126]. *P. falciparum*-infected erythrocytes can lead to various symptoms, such as ECG changes, myocarditis, tachycardia, pericardial effusion, and acute heart failure in patients with malarial infections [127,128]. Repeated infection by the *Plasmodium* parasite in endemic areas is reported to enhance angiotensin II and sphingosine-I phosphate (S1P), leading to hypertension and endothelial dysfunction [129,130]. A prospective study conducted by Kingston et al., with a sample size of 45 patients with either severe or acute *P. falciparum* infection, was found to be significantly associated with reduced cardiac index reserve and hypovolemia, as assessed by transthoracic echocardiography and invasive hemodynamic monitoring. These findings suggest that malaria-induced hemodynamic alterations can contribute to the development of cardiovascular complications in affected patients [131]. *Plasmodium* infections are associated with an increased risk of cardiovascular disease and heart attacks. The pathogenesis of malaria-related cardiovascular complications involves the dysregulation of cytokine and NO production through GPI-mediated mechanisms, as well as hemodynamic alterations.

*Neurological disorder*: *P. falciparum*-mediated malaria is known to cause cerebral malaria, a severe disease that can result in neurological complications such as seizures, loss of consciousness, and coma [132]. The pathogenesis of cerebral malaria involves the sequestration of infected erythrocytes in the brain microvasculature and the resulting immune response, leading to endothelial damage, vascular leakage, and hypoxia. These pathological changes can ultimately lead to neurological dysfunction and contribute to the high mortality rate associated with cerebral malaria [133]. Gene expression profiling studies have identified shared pathways and molecular signatures between cerebral malaria and neurodegenerative diseases, suggesting potential common mechanisms. The overexpression of the SNCA gene, which encodes alpha-synuclein, was observed in children with cerebral malaria. Alpha-synuclein aggregation is known to cause Parkinson’s disease, a neurodegenerative disorder that results in brain dysfunction. This suggests a potential link between the molecular pathogenesis of cerebral malaria and Parkinson’s disease [134]. A 53-year-old male diagnosed with cerebral malaria after traveling from Uganda later exhibited symptoms of neurodegenerative disease. The patient initially experienced prolonged seizures but later presented with ischaemic lesions associated with Parkinsonism. This case highlights the potential long-term neurological complications of malaria infections and the need for the continued monitoring of patients even after treatment for the acute infection. The mechanisms underlying the link between cerebral malaria and Parkinsonism remain unclear. Still, some studies have suggested that the aggregation of alpha-synuclein, a protein involved in Parkinson’s disease, may also play a role in the pathogenesis of cerebral malaria. Further research is needed to elucidate the precise mechanisms involved and to identify potential therapeutic targets for these debilitating conditions [135]. The overexpression of the PTEN-induced kinase I (PINKI) gene, which is involved in the clearance of dysfunctional mitochondria, was observed in subjects with cerebral malaria. This gene has also been implicated in the development of neurodegeneration and neuroinflammation, which are correlated with mitochondrial dysfunction and reported in several diseases, such as Alzheimer’s disease and multiple sclerosis. The findings suggest that cerebral malaria could induce mitochondrial dysfunction, which may contribute to neurodegenerative complications [136]. The HSPA1A gene encodes heat shock protein 70, which acts as a molecular chaperone and prevents protein aggregation by preventing misfolding. Overexpression of the HSPA1A gene has been observed in children with cerebral malaria and patients with epilepsy. The upregulation of HSPA1A mRNA expression is thought to result from inflammation and stress caused by brain injury. These findings suggest a potential link between the protective role of HSPA1A in preventing protein aggregation and the pathogenesis of neurological disorders associated with malaria and epilepsy [137]. Post-malaria neurologic syndrome (PMNS) is a rare neurological syndrome that occurs after a symptom-free period of acute malaria and is often misdiagnosed or unreported. Two hypotheses have been postulated to explain the development of PMNS: the ischemic hypothesis and the cytokine storm hypothesis. The ischemic hypothesis suggests that PMNS might result from the cytoadherence of *P. falciparum* and the blockage of brain microvasculature, leading to ischemic damage in the brain [138].

On the other hand, the cytokine storm hypothesis suggests that inflammatory cytokines such as IL-2 and IL-6 could damage the central nervous system by causing neuroinflammation and oxidative stress [139]. Malaria infection has been found to reduce the production of L-arginine and nitric oxide [140]. Nitric oxide is an important molecule that regulates endothelial cell function, including suppressing adhesion receptors. Low levels of nitric oxide in the system may contribute to the increased cytoadherence of Plasmodium-infected erythrocytes [141]. Although PMNS and autoimmune encephalitis converge in the symptom-free interval and after treatment with corticosteroids, no autoantibodies have been reported against malaria [141]. Neurocognitive decline, seizures, peripheral neuropathies, and tremors are mostly reported as clinical outcomes of PMNS [142]. Cerebral malaria, a severe and life-threatening complication of malaria, is commonly observed in sub-Saharan Africa and is often linked with epileptic seizures [143]. Studies have reported that some pediatric survivors of cerebral malaria have developed seizures and epilepsy within two years of the initial infection, highlighting the long-term neurological consequences of the disease [144].

*Renal disorders*: As per the literature, malaria has been associated with a rapid deterioration of the kidney. *Plasmodium* infection can lead to renal disorders due to the cytoadherence of infected red blood cells in the kidney vasculature, resulting in tissue damage and reduced kidney function [145]. Additionally, the immune response to the infection can cause inflammation and damage to the kidney. Certain antimalarial drugs can also cause kidney damage as a side effect. Therefore, it is important to monitor kidney function in patients with *Plasmodium* infection, especially those with pre-existing kidney conditions [146,147]. A retrospective study found that 5% of malaria patients with induced acute kidney injury (AKI) developed chronic kidney failure [148]. Patients with severe malaria are known to produce higher levels of proinflammatory cytokines [149]. A study conducted by Sinnai et al. on C57BL/6 mice infected with the murine malaria parasite *P. berghei* showed elevated levels of proinflammatory cytokines, establishing the potential roles of cytokines in kidney-related disorders [150]. Apart from this, endothelial activation induced the sequestration of RBCs, leading to impaired renal flow, and increased leukocyte infiltration may reflect the pathogenesis in AKI [151]. Autoantibody formation leads to immune-complex-mediated complement activation and may cause renal injury in malaria patients [152]. Monitoring kidney function is crucial, as severe malaria may increase proinflammatory cytokines and endothelial activation, contributing to the pathogenesis of acute kidney injury.

### 3.3. Dengue Virus and Associated Immune Evasion Strategies

#### 3.3.1. Viral Sensing by the Host’s Immune System

DENV infection is a growing global burden, affecting countries regardless of economic status. DENV exists in four serotypes (DENV 1-4), all of which have the potential to cause severe conditions. These four serotypes facilitate different clinical manifestations ranging from mild symptomatic to asymptomatic. Severe forms are more likely to manifest in those who have been exposed to a secondary DENV infection of a different serotype. Additionally, there is a higher risk of developing dengue shock syndrome (DSS) and antibody-dependent enhancement (ADE) post-secondary infection by heterogenous dengue serotypes. ADE occurs when non-neutralizing antibodies from a previous infection bind to a different serotype, facilitating viral entry into immune cells and leading to increased viral replication and disease severity. This means that the immune response to a second infection with a different serotype can be stronger and more damaging than the response to the first infection [153].

The successful transmission of the dengue virus commonly activates several immune responses within the host’s body. This includes the recognition of pathogen-associated molecular patterns (PAMPs) by the pattern recognition receptors (PRRs) of our innate immune system [154]. Toll-like receptor-3 (TLR3), TLR-7, retinoic acid-inducible gene I (RIG-I), and melanoma differentiation-associated protein 5 (MDA5) are among the pattern recognition receptors (PRRs) that induce an antiviral state by activating cytokine and chemokine production [155]. The RIG-I-like receptor pathway (RLR) primarily detects viral nucleic acid through the cytosolic sensors RIG-1/DDX58, MDA5, and Laboratory of Genetics and Physiology 2 (LGP2 or DHX58). Upon activation, RIG-I and MDA5 interact with mitochondrial antiviral-signalling protein (MAVS) through the caspase activation and recruitment domain, which activates IκB kinase ε (IKKε), TANK-binding kinase-1 (TBK1), and phosphorylates IFN regulatory factors (IRF3, IRF7), leading to the production of type I interferon, mainly IFN-β [156,157]. Additionally, viral RNA is recognized by TLR3 and converges into MAVS, stimulating IRF3, IKKε, and IFN-β. The outer membrane of the mitochondria plays a vital role in initiating and amplifying the innate immune system [158].

**Figure 3:**
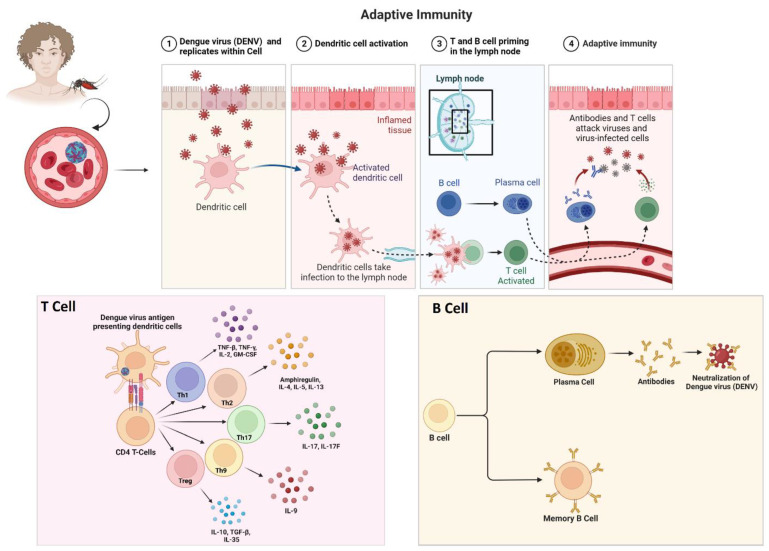
The adaptive immune responses combine forces to fight the dengue virus. B cells produce antibodies that specifically recognize and neutralize the foreign viral particles, and cytotoxic T cells recognize and kill cells that are infected with the dengue virus.

During DENV infection, the STING/cGAS pathway is activated by cyclic GMP-AMP synthase (cGAS), a secondary messenger molecule, which stimulates TBK1, phosphorylates IRF3, and facilitates the production of IFN-I [159]. This pathway recognizes DNA molecules and is also essential in developing innate immune responses against viral RNA. DENV infection causes mitochondrial damage, releasing mitochondrial DNA (mtDNA) into the cytosol and activating the cGAS-STING pathway [160,161,162]. Recent studies show that mtDNA also activates TLR9, which recognizes nonmethylated CpG islands in dendritic cells [163]. Additionally, the inflammasome secretes interleukin (IL)-1β, inducing the release of mtDNA and activating the cGAS/STING pathway to produce IFN [164]. Thus, DENV infections trigger these two major pathways, RIG-I/MAVS and cGAS/STING, which need further study to understand their crosstalk (Figure 3).

#### 3.3.2. Immune Evasion Strategies of Dengue Virus Strains

The immune response to dengue virus infection is complex and can have different effects on the four virus serotypes [165]. When a person becomes infected with one of the serotypes, their immune system mounts a response to fight the infection [166]. This response includes the production of antibodies specific to the infecting serotype, which can provide lifelong immunity to that particular serotype. However, this immunity is only partial and temporary for the other three serotypes [167].

This increased viral replication can lead to a more severe form of the disease, such as dengue hemorrhagic fever (DHF) or dengue shock syndrome [168]. DHF is characterized by bleeding, plasma leakage, and organ dysfunction, while DSS involves dangerously low blood pressure and can be fatal [169]. The risk of developing severe dengue is generally higher during secondary infections with a different serotype. However, the specific interactions between the immune system and the different serotypes are still not fully understood [170].

#### 3.3.3. Countermeasures to Hijack RLR Signaling

DENV RNA molecules have intrinsic factors that help evade and hijack the host’s innate immune system. The sensing interference mainly depends on two specific alterations: partial degradation by host nucleases and 2′-O-methylation. 2′-O-methylation imitates cellular mRNA, enabling it to evade the host’s immune barrier. Additionally, DENV RNA interferes with the activation of RIG-I by preventing the formation of a signaling complex, ultimately suppressing the production of type I interferon. Understanding these evasion mechanisms is crucial for developing effective antiviral strategies [171,172]. When DENV RNA is partially degraded by host nucleases, it generates a sub-genomic flavivirus RNA (sfRNA). The DENV sfRNAs are known to inhibit the deubiquitylation of TRIM25 by ubiquitin-specific protease 15 (USP15). TRIM25 is an important factor in RIG-I signaling, and its inhibition ultimately suppresses the production of type I interferon, allowing the virus to evade the host’s immune system. These findings suggest that sfRNAs play a crucial role in the pathogenesis of DENV infections [173]. The inhibition of TRIM25 by DENV sfRNAs hinders the polyubiquitylation of RIG-I and prevents its dimerization with the CARD domains, which is a crucial step in the activation of the RIG-I signaling pathway. This ultimately leads to the suppression of the interferon signaling process, allowing the virus to evade the host’s immune system. These findings highlight the significance of the interaction between viral RNA and host factors in the pathogenesis of DENV infections [174].

The NS3 of the DENV has a phosphomimetic RxEP motif that inhibits the translocation of RIG-I to the mitochondria by restraining 14-3-3ε [175]. The 14-3-3ε protein is a primary requirement for the association of RIG-I/TRIM25 and eventually interacting with MAVS in mitochondria [176]. A morphological change in the mitochondria has also been reported to modulate antiviral signaling [177]. The strategy of mitochondrial morphodynamics used by the DENV is not unique since similar strategies are used by other viruses, including severe acute respiratory syndrome-related coronavirus (SARS–CoV) and hepatitis virus [178]. Non-structural proteins of DENV, including NS2A, NS2B, and NS4B, block the phosphorylation of IRF3 by inhibiting the kinase activity of IKBKE and TBK1 [179].

#### 3.3.4. Countermeasures to Hijack the cGAS/STING Pathway and IFN Signaling

After DENV infection, IFN induction is triggered by activating the cGAS/STING pathway by releasing mtDNA in the cytoplasm [159]. The NS2B protein of DENV identifies cGAS for lysosomal degradation. On the other hand, the protease activity of the NS2B-NS3 complex cleaves STING, thereby inhibiting the production of type I IFN at an optimal level [162,180]. During DENV infection, the JAK-STAT signaling cascade is downregulated by NS4B, specifically by hijacking STAT1 activation by an unknown mechanism yet to unfold [181]. It is hypothesized that it prevents STAT1 activation through dephosphorylation or by degrading the activated STAT1. In addition, the NS5 protein attaches to STAT2, thereby decreasing its expression level and ultimately preventing the normal function of the JAK-STAT pathway [182].

#### 3.3.5. Role of microRNA in DENV Pathogenesis

MicroRNA (miRNA) plays a critical role during viral infection as viruses use these miRNAs to hijack the host’s immune system and facilitate viral replication. A microarray analysis study was carried out using a blood sample of a patient with DENV infection, expressing 348 miRNAs after DENV infection [183]. Another patient’s serum infected with DENV-1 was analyzed, and it was found that 12 miRNAs were downregulated and 41 miRNAs were upregulated [184]. Previously, another study found that miR-21 was significantly increased after infection by DENV-2 in HepG2 cells. However, the exact mechanism by which miR-21 facilitates the replication process is unclear, but it can be rationalized that it might target the NS1 protein of the DENV [185,186]. Another midgut-specific miRNA, miR-281, was expressed after DENV-2 infection in C6/36 cells. It was observed that miR-281 promotes viral infection by targeting the 5′-UTR of the viral genomic RNA, thereby enhancing the replication process of DENV [184].

In both human peripheral blood and primary monocytes, an increase in miRNA, specifically miR-146a, was detected upon infection with DENV. This miRNA was found to suppress the production of IFN-β by binding to tumor necrosis factor receptor-associated factor 6 (TRAF6) [187]. The TLR signaling pathway is enabled by TRAF6 and interleukin-1 receptor-associated kinase (IRAK1), which miR-146a targets. As a result, the production of IFN-I is diminished, ultimately allowing the pathogen to avoid being attacked by the host’s immune system [188]. This study confirms that neutralizing miR-146a restores the optimal IFN-I production level. Another miRNA, namely miR-378, hinders the production of GrzB in the NK cells and thus promotes DENV replication [189].

#### 3.3.6. Dengue Fever and Its Association with Other Disease Outcomes

*Cancer*: A nationwide population-based cohort study has been published correlating the risk of developing leukemia with preceding dengue virus infection [190]. The observation of abnormal hematologic profiles and bone marrow suppression in leukemia patients led researchers to question whether there is a connection between dengue viral infection and the onset of leukemia. Infected patients’ bone marrow has also yielded dengue virus, and research suggests that hematopoietic progenitor cells are particularly vulnerable to dengue viral infection [190]. Dengue fever was determined to be present in 28 different patients. A total of 9 (32.14%) patients had hematological malignancies, leaving 19 (67.85%) patients with solid tumors. In 23 patients (82.14%), chemotherapy was still being administered, and 5 patients (17.85%) were being monitored [191].

*Cardiovascular diseases*: DENV-infected patients are found to have higher levels of proprotein convertase subtilisin/Kexin type 9 (PCSK9) in their plasma [192]. PCSK9 has also been reported to be associated with coronary artery disease, particularly with myocardial infarction resulting due to the rupture of the plaques [193]. In a study by Miranda et al., 12 out of 81 subjects had elevated cardiac biomarkers during dengue infections [194]. DENV could infect myocardial tissue and myocytes, thus altering calcium metabolism and causing myocarditis in pediatric patients [195]. ECG abnormalities, such as ST segment changes, and bradyarrhythmia, including atrioventricular block ventricular tachycardia, are reported [196,197]. Chaturvedi et al. experimentally proved that cardiac injury follows DENV infection [198].

**Figure 4:**
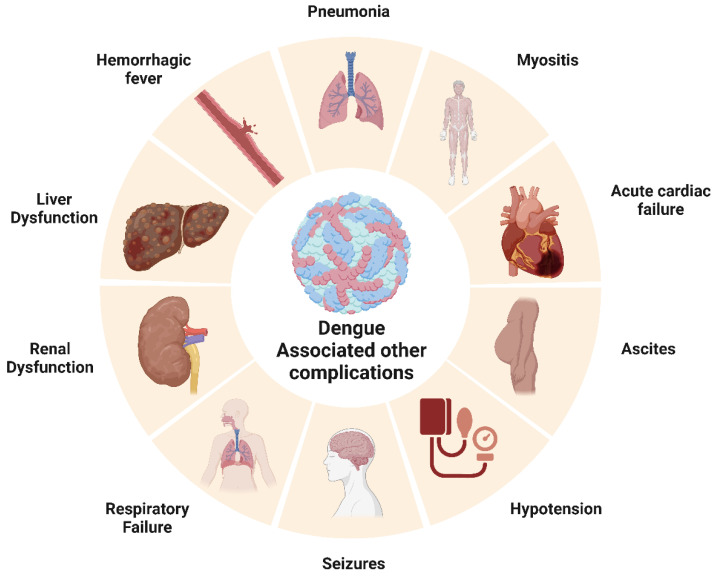
Dengue and its link with other disease complications after dengue virus infection and disease severity.

*Neurological disorders*: In 2009, the World Health Organization (WHO) categorized several neurological disorders, including encephalopathy, encephalitis, dengue muscle dysfunction, and neuro-ophthalmic disorders, as potential complications resulting from severe dengue infection. A case study conducted in Brazil involving 150 patients with severe dengue infections reported diverse neurological symptoms. Additionally, DENV particles were detected in 48.8% of dengue-positive cases, thus providing further evidence linking neurological changes with DENV infection. Clinical manifestations of infection, such as meningitis (19.5%), encephalitis (46.3%), and meningoencephalitis (34.1%), were common [199]. The neuro-pathogenesis of DENV infection can be correlated with the presence of viral particles in cerebrospinal fluid (CSF) and damage to the blood–brain barrier (BBB) [200]. Dengue viruses are also reported to infect various immune cells and murine neuronal cells directly [201]. Recent studies have suggested the role of DENV NS1 antigen in triggering macrophages via TLR4, leading to the release of proinflammatory cytokines such as TNF-α, IL-12, and IL-4 and damaging the blood–brain barrier [202]. Strokes associated with DENV infection, either ischemic or hemorrhagic, are uncommon but not absent. Hemorrhages result from plasma leakage and vasculitis, but the bleeding is mainly intracranial [203]. Several studies also reported parkinsonism associated with DENV infection, mainly after recovery from acute illness. Patients developed symptoms such as bradykinesia, bradyphonia, and cogwheel rigidity, which could result from immune-mediated reactions [204]. In a prospective case-control study on 5400 children admitted with dengue hemorrhagic fever in Vietnam, 27 showed features of neurological syndromes. Seizures were reported in nearly 77% of individuals with clinical neurological manifestations [205]. Autoimmune conditions of neuromyelitis optica spectrum disorder (NOSD) were reported in two dengue-infected patients with aquaporin 4 antibodies in their serum [206]. Similarly, autoimmune conditions such as acute disseminated encephalomyelitis (ADEM) are also found among dengue-related neurological disorders. The most common manifestation was altered consciousness, followed by seizures and vision problems. The pathophysiology of this autoimmune reaction is unknown, but an auto-reactive attack on myelin could be due to imbalanced T cell tolerance [207] (Figure 4).

*Renal disorders*: Acute kidney injury in DENV-infected patients is considered to be an expanded dengue syndrome. A study by Nair et al. using the KIDGO criteria reported 69.4% dengue-associated AKI patients out of 85 subjects [208]. The correlation between dengue hemorrhagic fever (DHF) and kidney damage was studied with kidney specimens from fatal DHF patients. More sickle erythrocytes were located in glomeruli and inflammatory infiltrates. High numbers of IL17 and IL18^+^ showed the possibility of local lesions and enhanced vascular permeability [209]. Urine abnormalities were also diagnosed in DENV-infected patients. Increased blood and protein levels were reported among hospitalized dengue patients in Australia [210]. Jayarajah et al. conducted a prospective study among 170 DHF/DF-positive patients and measured the urine albumin/creatine ratio. They found the mean ratio to be 177 ug/mL and concluded that there was a high occurrence of microalbuminuria in DENV-infected patients [211]. A rare case where DENV infection was followed by the autoimmune conditions of lupus and nephritis has been documented in Maharashtra, India. Upon the microscopic evaluation of a kidney tissue biopsy, glomerulonephritis and segmental sclerosis (Stage IIIC) were observed. The possible reason for lupus nephritis can be described as a classic immune complex-mediated autoimmune reaction [212]. Hence, dengue-triggered immune reactions could form pathogenic autoantibodies against host neutrophils and the glomerular basement membrane [213].

### 3.4. Zika Virus and Associated Immune Evasion Strategies

Zika was first identified in Uganda and has since expanded extensively into the Americas and Western Pacific, with 86 countries that have reported active ZIKV by 2019 [214]. During pregnancy, Zika virus infection may cause congenital disorders such as CNS malformations, microcephaly, cerebral palsy, and encephalitis in fetuses and neonates, leading to brain anomalies [215]. The Zika virus employs various strategies to invade the vertebrate immune response and cause disease. Here, we summarize some of its immune evasion tactics.

The ZIKV structural E protein is responsible for the ability of the virus to bind to a different receptor and simultaneously inhibit IFN production [216]. Viral genome and sub-genome RNAs inhibit IFN signaling. An experimental approach involving the temporary production of ZIKV-3’ UTR and the transfection of RIG-I or MDA-5 agonists revealed that ZIKV sfRNAs obstruct RIG-I- and MDA-5-mediated IFN induction [217]. ZIKA-infected cells show a significant reduction in the expression of IFN and ISG. The viral proteins NS1, NS4A, and NS5 have been identified as major inhibitors of type 1 IFN. Additionally, it has been observed that the amount of antiviral transcriptional activator is decreased in ZIKAV-infected cells due to STAT2 degradation by NS5.

#### 3.4.1. Hijacking the RNA Interference (RNAi) Pathway

Zika virus alters the RNAi pathway, thereby suppressing RNA silencing. In mouse brain tissue and hNSCs (human neuronal stem cells), ZIKV infection upregulated miR-124-3p and let-7c, which downregulated transferrin receptor (TFRC) and HMGA2 (high-mobility group AT-hook 2) mRNAs, respectively [218]. Additionally, ZIKV infection elevated miR-125a-5p and miR-125a-3p, exhibiting inhibitory effects on MAVS, a vital component of the innate immune system’s RIG-I and type I IFN response pathways [219].

#### 3.4.2. Zika Virus Escapes NK Cell and DC Detection

Zika virus infection is detected by RIGI and IRF3, which cause the release of type I IFN and the overexpression of MHC class I and CEACAM1. MHC I expression can be dramatically decreased by blocking IFN-beta [220]. Through this IFN-beta-blocking mechanism, the Zika virus can escape NK cell detection. DCs are essential for detecting viral infections and coordinating both immediate and long-term antiviral responses. ZIKV promotes the expression of lipid metabolism genes by increasing SREBP TF binding and transcriptional initiation. By lowering the amount of cellular cholesterol, SREBP2 suppression by medication or genetics reduces the amount of ZIKV infection in monocyte-derived DCs (moDCs), possibly due to the reduction of ZIKV infection during the replication, assembly, and/or budding phases. To infect human moDCs, ZIKV thus encourages the activation of SREBP2-dependent cholesterol production, providing a unique treatment approach for ZIKV [221].

#### 3.4.3. Immune Evasion Strategies for Various Zika Virus Strains

Despite being a single serotype, genetic diversity among different strains of ZIKV can lead to variations in surface proteins, helping the virus evade neutralizing antibodies and the host’s immune system [222]. Additionally, ZIKV can infect cells in immune-privileged sites such as the placenta, testes, and central nervous system, avoiding immune surveillance and establishing persistent infections [223]. ZIKV can also suppress host cell apoptosis, maintaining a cellular environment conducive to viral replication and effectively evading immune defenses [224]. The virus can hijack the autophagy pathway, a cellular process responsible for maintaining homeostasis, thus promoting its replication and evading the host’s immune response [225].

#### 3.4.4. Zika Virus Infection and Its Association with Other Disease Outcomes

*Cardiovascular diseases*: Although mosquito-borne Zika infection is mainly associated with neurological syndromes, myocarditis was found to be associated with Zika infection owing to increased troponin I and creatine phosphokinase [198]. Brasil et al. reported a potential association of ZIKAV infection with congenital heart disorders in infants [226]. In a recent study, Rashid et al. reported that ZIKAV infection of human Sertoli cells significantly altered several proteins involved in cardiac hypertrophy [133].

*Neurological disorders*: ZIKV is known to preferentially infect a wide range of neural cells, including neural stem cells (NSCs), oligodendrocyte precursors, astrocytes, and microglia [227]. Symptomatic Zika infection is mainly restricted to mild and self-limiting febrile disease, but recent shreds of evidence link Zika to microcephaly in fetuses. Microcephaly can be classified into primary and secondary microcephaly. The former is characterized by small brain size owing to defects during embryonic development, while the latter reflects normal embryonic development, but brain damage restricts brain development [228,229]. A case–control study conducted in eight hospitals in Brazil from January to May 2016 linked microcephaly and Zika virus infection. This study analyzed cerebrospinal fluid sampling and performed an RT-PCR analysis of 32 cases and 64 controls. They found that 41% of neonates with microcephaly were positive for Zika infection, whereas the rest were uninfected. Zika virus was reported to infect neural progenitor cells (NPCs), and the increased number of viral particles damaged the cells, causing cellular apoptosis and leading to transcriptional attenuation [230]. The dysregulation of normal cellular functions in NPCs could lead to developmental defects. In organoid models, Zika virus infection was associated with a reduced number of neural progenitor cells. TLR-3 was reported to be upregulated in this model, and organoid shrinkage was observed. TLR-3 was thought to be a potential trigger for cellular apoptosis and thought to inhibit neurogenesis after Zika infection [230]. Activation of TLR-3 by an RNA virus mimetic poly (I: C) was found to modulate NDMA receptors and was associated with impaired neurodevelopment and abnormal arrangements of synaptic proteins [231]. A potential clinical link between ZIKV infection with autism spectrum disorder (ASD) was also reported in Brazil. The child in this case study was positive for Zika IgG, and ASD was diagnosed by neurologists and confirmed by the Diagnostic and Statistical Manual of Mental Disorders (DSM-5) and Autism Spectrum Quotient (ASQ). Although exome sequencing did not reveal any pathogenic variant in genes related to ASD, the possibility of in utero infection by ZIKV must be clinically considered [232]. Epilepsy surveillance using a questionnaire and video electroencephalography in a cohort of normocephalic and ZIKV-exposed children and unexposed controls suggested a modest occurrence (IR: 2.8%, 95% CI: 0.34–9.81%) of epilepsy in exposed subjects. Although this study has a small sample size and low incidence of epilepsy, the neurodevelopmental assessment of children with suspected or confirmed in utero ZIKV infection should consider epilepsy surveillance during their first two years of life [233]. Kung et al. profiled the transcriptome of primary neuron cultures infected by ZIKV and used unbiased gene ontology enrichment analysis and in vivo confirmation in an immunocompetent mouse model. An altered expression of genes associated with bipolar disorder and schizophrenia was reported. In vivo validation using mice suggested that TNF-α signaling is a major switch for ZIKV-induced neurologic gene expression [234]. A probable link between neurodegenerative Alzheimer’s disease (AD) and ZIKV infection was studied using brain organoids. An upregulation of key Alzheimer’s pathologies, including amyloid beta (Aβ) and p-tau, was noticed in both AD and WT organoids exposed to ZIKV infection. The mechanism of neurodegenerative pathology was identified as a stress response in the endoplasmic reticulum and subsequent activation of the PERK-eIF2α axis [235]. Inhibition of PERK-mediated signaling reduced neurodegenerative phenotypes, including Aβ and p-tau [236]. In addition to its direct effect in causing neuropathologies, ZIKV infection is also associated with epigenetic alteration in the brain. Janssens et al. reported the altered DNA methylome of NPCs, astrocytes, and differentiated neurons in an organoid model. However, the mechanism is not well defined, and the question remains whether viral infection dysregulates the epigenetic regulators before any epigenetic shift [237]. The autoimmune condition Guillain–Barre syndrome (GBS) is also correlated with ZIKV-infected patients. In a case–control study of patients experiencing Guillain–Barre syndrome, 41 (98%) out of 42 patients were found to have anti-Zika IgG or IgM antibodies [238]. This may be due to a possibility of molecular mimicry where ZIKV cross-reacts with some neurological proteins and leads to an autoimmune catastrophe [239].

**Figure 5:**
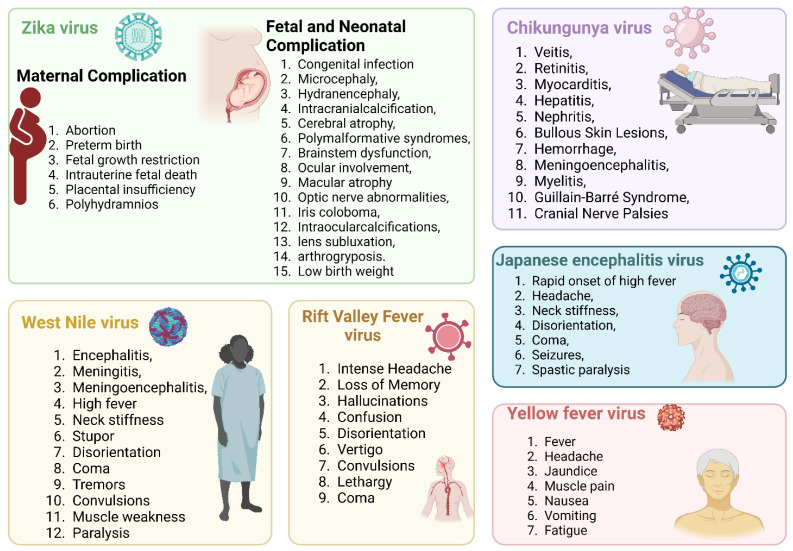
Mosquito-borne viral diseases (West Nile, chikungunya, Zika, yellow fever, Japanese encephalitis, Rift Valley fever) are recognized as multi-organ diseases with a broad spectrum of manifestations. Post-acute viral syndromes persist, presenting with prolonged effects with post-disease multiorgan complications.

*Renal disorders*: The incidence of ZIKV infecting renal proximal tubular epithelial cells was demonstrated by Chen et al. using a C57BL/6 mouse. The authors found the glomerulus to be more infected and reported swelling of kidneys and apoptosis of renal cells [240]. Liu et al. reported that ZIKV infection causes AKI in mouse models by analyzing AKI-related biomarkers such as serum creatine kinase, kidney injury molecular-1 (Kim-1), and neutrophil gelatinase-associated lipocalin (NGAL). They proposed a mechanism where infection triggered the inflammasome’s Nod-like receptor 3 (NLR3) and apoptosis by suppressing Bcl-2 [241]. Congenital Zika syndrome patients were also found to be associated with a neurogenic bladder. The overactive bladder was evident in 21 of the 22 individuals evaluated, along with decreased bladder capacity and increased detrusor filling pressures [242] (Figure 5).

### 3.5. Chikungunya Virus and Associated Immune Evasion Strategies

CHIKV infection is responsible for long-term febrile illness, arthritis, and other complications such as chronic synovitis due to its persistence in joint-associated tissues [243]. CHIKV has acquired several mechanisms to hijack the host’s immune system. CHIKV non-structural protein-2 (NSP2) regulates the IFN-induced JAK-STAT signaling pathway [244]. CHIKV infection antagonizes the antiviral IFN response by creating mutations in the KR649AA site in the NLS of NSP2 or redirecting the NSP2 C-terminal methyltransferase-like domain into the nucleus [245]. Recent findings demonstrate that the NSP2 of VEEV specifically interacts with the nuclear importin molecule karyopherin α-1 (KPNA1) and downregulates STAT1-dependent type I and II IFN signaling [246]. The co-expression of MDA5/RIG-I along with NSP2, E1, and E2 inhibits more than 80% of the MDA5/RIG-I mediated IFN-beta promoter activity in the presence of viral protein [247]. Bone marrow stromal antigen 2 (BST-2), a well-characterized antiviral IFN stimulatory gene (ISG), prevents viral release. Upregulation of NSP1 counteracts the effect of BST-2, thereby enabling the release of viral particles from infected cells [248]. CHIKV can evade immune surveillance by suppressing the expression of zinc-finger antiviral protein (ZAP), an IFN-induced antiviral factor [249].

Virus replication can be inhibited by the transcription and expression of the cGAS-STING innate immune pathway. The CHIKV NSP1 either inhibits IFN-β promotor activation by interacting with STING or degrades cGAS to stabilize the viral protein [250]. CD8^+^ T cells are critical for the clearance of viral infection from lymphoid tissue but not joint-associated tissue. In contrast to humoral immunity, its role is poorly understood. Therefore, different studies suggest that CHIKV establishes and maintains a persistent infection in joint-associated tissue by evading CD8^+^ T cell immunity, but the underlying mechanism involved is still unclear [243]. The major immune checkpoints reported in CHKV infection are IFN-a, IL-10, and CCL2 [251]. RNAi is one of the major antiviral defense mechanisms present in eukaryotes. NSP2 and NSP3 are the two non-structural proteins that exhibit RNAi suppressor activity by separating double-stranded RNAs [252]. Other studies believe the capsid protein interacts with the viral RNA to prevent their interaction with RISC, which contributes to the inhibition of RNA-mediated viral RNA degradation [253].

#### Immune Evasion Strategies of Various Chikungunya Virus Strains

Immune response mechanisms to chikungunya virus infection are generally conserved across different strains. The genetic differences between the strains can influence virus transmission, spread, and virulence [254]. However, the immune response to CHIKV infection is primarily directed against the conserved viral antigens shared among different strains [255].

Nevertheless, the unique characteristics of each genotype can affect the disease’s epidemiology and severity [256]. The West African genotype originates in West Africa and has no major adaptive mutations identified. It is characterized by limited outbreaks, mainly occurring within the sylvatic cycle (mosquitoes-primates-mosquitoes). The ECSA (East/Central/South African) genotype is found in Eastern, Central, and Southern Africa, as well as the Indian Ocean islands. It has an A226V mutation in the E1 glycoprotein, which increases transmission by *Aedes albopictus*. This genotype is responsible for major outbreaks in the Indian Ocean islands, India, and Southeast Asia and is associated with both urban and sylvatic cycles. The Asian genotype is prevalent in Asia and the Pacific, with no major adaptive mutations identified, but it has caused major outbreaks in the Pacific region between 2013 and 2014. This genotype is associated with an urban cycle (mosquitoes-humans-mosquitoes) [257].

### 3.6. Yellow Fever Virus and Associated Immune Evasion Strategies

**Figure 6:**
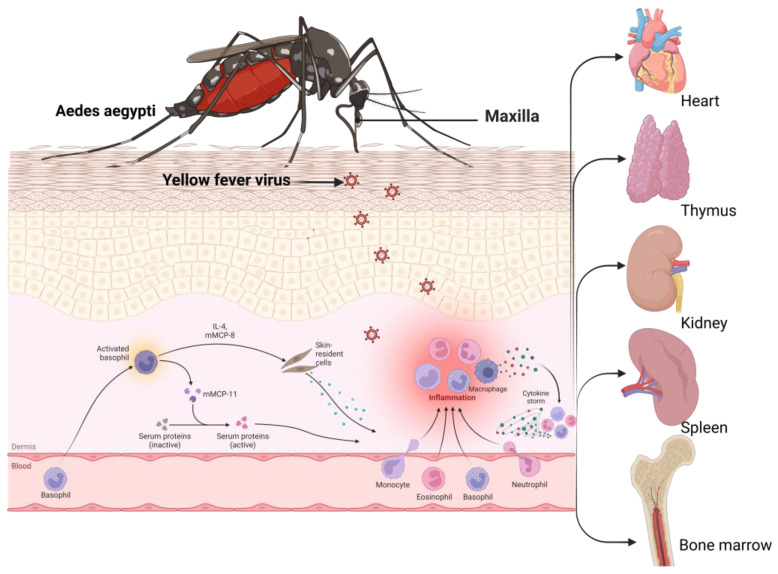
Representation of yellow fever (YF) pathway development. This figure illustrates the sequence of events in YF-related disease development, starting with the bite of an infected mosquito and ending with apoptosis and a heightened inflammatory reaction. The presence of the yellow fever virus (YFV) is indicated by a distinct color. It is hypothesized that intense viral replication in the liver sets off a series of molecular reactions, leading to considerable disruption in cytokine balance and an increased release of pro-inflammatory substances. Consequently, this results in substantial vascular impairment and multi-organ malfunction.

YFV, the causative agent of yellow fever, is a prototypic member of the genus *Flaviviridae* and is known to cause one of the most severe infectious diseases [9]. A wide range of clinical symptoms is associated with YFV infection, ranging from entirely asymptomatic hemorrhagic fever to a high mortality rate [258]. After disease remission, 20–60% of patients went through a more lethal stage of the illness, marked by hemorrhagic fever, jaundice, thrombocytopenia, liver failure, and renal failure [11]. In the liver, viral replication plays a major role in disease establishment (Figure 6).

YFV has evolved several immune evasion mechanisms, including hepatocyte apoptosis, to manifest infection, which is discussed briefly. MDA5 and LGP2 constitute part of the RIG-1-like receptor family. Upon the recognition of intracellular RNA by pattern recognition receptors, namely RIG-1, an interferon response to infection is mediated [259]. RIG-1 generally recognizes dsRNA with triphosphate and U or A-rich motifs [260]. Long dsRNA is mainly recognized by MDA5 [260]. YFV infections are restricted by both RIG-1 and MDA5, which play important roles in mediating antiviral effects [261]. The cap 1-2′O-methylation of RNA prevents RIG-1 recognition [262]. This genomic alteration helps in viral immune evasion and survival inside the host.

When STAT1 is phosphorylated via type I IFN signaling, NS5 (non-structural) protein binds with STAT2; this binding and subsequent suppression of IFN signaling is mediated by the E3 ubiquitin ligase TRIM23, which polyubiquitinates NS5 [262]. In this way, YFV represses IFN signaling and blocks the antiviral effects of IFN-1 [263]. YFV actively replicates inside the liver and is responsible for hepatocytotoxicity. The pathogenesis of yellow fever is mediated by the apoptosis of hepatocytes [11]. The disease progression is maintained by a systemic and unbalanced cytokine storm [264]. CD4^+^ Th1 and Th3 cells have been noticed in livers infected with YFV [265,266]. The CD4^+^ T cell-expressed TNF-α cytokines mediate hepatic damage and CD8^+^ T cell cytosis [10]. Accordingly, Th3 CD4^+^ T cells express the pro-apoptotic cytokine TGF-β, an anti-inflammatory protein and a pro-apoptotic inducer [265,266]. The generation of TNF-α and TGF-β indicates that the immunopathogenesis of YFV may involve an imbalanced pro- and anti-inflammatory cytokine response [267]. This cytokine imbalance in the liver displays immune evasion mechanisms, leading to the progression of the viral infection inside the human host.

#### Immune Evasion by YFV Strains

Currently, there are limited data on how the different genotypes of YFV affect immune responses in humans. However, the immune responses to YFV generally seem to be consistent across genotypes [268]. This is evidenced by the fact that the yellow fever vaccine, based on the live-attenuated 17D strain, has been effective against all YFV genotypes [269].

The immune system combats YFV through both innate and adaptive immune responses, as mentioned earlier. While the specific immune response may vary slightly among genotypes due to genetic differences, these variations are not well understood and are likely to be minor [270]. Despite genetic differences among the YFV genotypes, the immune response is generally consistent across genotypes. Individual factors can influence the severity of the disease and the effectiveness of the immune response, but these are likely unrelated to the specific genotype of the virus. More research is needed to understand the potential variations in immune responses to different YFV genotypes.

### 3.7. Rift Valley Fever Virus and Associated Immune Evasion Strategies

RVFV primarily affects domestic animals and occasionally humans, with an intermediate mortality rate. Mosquitoes transmit the virus, and outbreaks are most associated with periods of heavy rainfall and flooding, which create ideal breeding conditions for the mosquitoes that carry the virus. While most human infections are mild or asymptomatic, in rare cases, RVFV can cause severe disease, including hemorrhagic fever and encephalitis [271]. The entry of the RNA virus into the host is followed by a complex set of innate immune responses [272]. Retinoic acid-inducible gene I (RIG-I), melanoma differentiation factor 5 (MDA5), and Toll-like receptors (TLRs) are among the pattern recognition receptors (PRR) that recognize this virus [273]. RIG-I also triggers an interferon response inside the host cell and maintains an antiviral state [272]. The RVFV has developed sophisticated strategies to evade the immune system and dysregulate the immunological pathways to successfully initiate infection and manifest into a clinical condition, which will be discussed briefly.

The post-transcriptional alternative splicing mechanism is altered by this viral invasion [274,275]. The progression of RVFV inside the host is inhibited by RIO kinase 3 (RIOK3), which plays a significant role in the production of IFN-1 through PRR signaling mediated by RIG-I-like receptors [276]. The antiviral effects of RIOK3 are stably maintained by the interaction of TRA2-β with fixed regions of RIOK3 pre-mRNA, which promotes the constitutive splicing of RIOK-3 mRNA [277]. Upon viral infection, TRA2-β is alternatively spliced, decreasing its cellular levels, forcing the RIOK-3 mRNA to undergo alternative splicing and produce the variant isoform RIOK-3 X2 [278]. This spliced isoform RIOK-3 X2 lowers the expression of interferon and helps in the successful evasion of the host’s immune pathways [277]. RIOK-3 X2 also plays a role in increasing the inflammatory NF-κβ response [272].

#### 3.7.1. The Role of RVFV Non-Structural Proteins in Immune Evasion

The S segment of the RVFV genome encodes the non-structural proteins, which can suppress the immune system [279]. NSs form a complex with Sin3A-associated protein 30 (SAP30) and transcription factor Yin Yang 1 protein (YY1) and suppress the activation of the IFN-β promoter [280]. NSs also contribute to viral evasion by preventing the IFN-β promoter from being activated. NSs mediate the inhibition of host transcription and the activation of viral translation. Eukaryotic transcription factor IIH helps in the initiation of transcription by eukaryotic RNA polymerase II. The core complex of TF IIH comprises XPD, XPB, p44, p62, p8, p34, and p52 [272]. RVFV NSs can competitively bind to p44 and prevent it from binding with XPD [281]. P62 is also degraded by the ubiquitin–proteasomal pathway, where NSs work as an adaptor protein in the cullin 1-Skp1-FBXO3 E3 ligase complex [282,283]. The host transcription is halted by these two mechanisms, which affects the recruitment of the TF IIH complex in the nucleus.

RVFV-infected cells have shown nuclear deformities such as micronuclei and lobulated nuclei mainly due to defects in chromosomal cohesion and segregation [284]. These damages occur due to the accumulation of NSs filaments inside the nucleus [272]. The filaments also play a key role in arresting the cell cycle at the S or G0/G1 phases [285]. The chromatin DNA cohesion and segregation are affected by the SAP30-YY1 complex formed by NSs proteins [272]. Therefore, RVFV impairs the host cell replication machinery via NSs-mediated DNA damage. From the recent findings, it can be said that the virus has established effective immune evasion mechanisms to counteract the host’s immune response and has also developed ways to attack various cellular processes and pathways, which aids in the successful manifestation of infection and contributes to viral pathogenicity.

#### 3.7.2. Immune Evasion by Different RVFV Strains

RVFV strains evade the immune system through various mechanisms, including antigenic variation, inhibition of the interferon response, modulation of host cell machinery, immune evasion proteins, and suppression of dendritic cell function. Different strains use different combinations of these strategies, making developing a single, effective vaccine or treatment challenging. Ongoing research aims to uncover immune evasion mechanisms to create targeted therapies and control outbreaks [272].

### 3.8. Japanese Encephalitis Virus and Associated Immune Evasion Strategies

JEV is known for its single-stranded RNA genome, which mutates faster and spreads through mosquitoes. Although the immune sentinels of the host are always ready to shoot any invading JEV, the latter has smartly evolved to rule out the host’s defense. Invasion of JEV triggers the RIG-I/IRF-3 and PI3K/NF-kB axis, facilitating the release of inflammatory cytokines and chemical cues that trigger more immune cells [286]. JEV was found to activate cellular autophagy, which eventually promoted its survival but downregulated the innate antiviral response. JEV-infected cells with inactivated autophagy produced more IFN [287]. A plausible reason could be the low clearance of mitochondrial antiviral signaling protein (MAVS) and its high aggregation, which switches INF3 and NF-kB [287]. Another strategy for blocking INF signaling was reported by Lin et al., where NS5 acts as a phosphatase and blocks INF-induced JAK-STAT signaling [288]. JEV NS1 also interacts with cyclin-dependent kinase 1 (CDK1) and prolongs the phosphorylated state, activating CREB. This CREB acts as a transcription factor, activates miR-22, and reduces INF-I production by targeting MAVS [289].

Immune evasion leading to reduced IgM and neutralizing antibodies against JEV was reported in a mouse model where virus infection elevated myeloid-derived suppressor cells (MDSCs). In this study, MDSCs were found to suppress the T follicular helper cell differentiation that brought down the splenic B cell population and plasma cells, leading to a shunted humoral immune response [290]. JEV also infects the DCs and impairs these antigen-presenting cells along with the production of IL-10, an anti-inflammatory cytokine. IL-10 production promotes JEV survival by reducing costimulatory activity and CD8^+^ T cell priming [291]. Similarly, JEV infection has been reported to modulate immune responses in a mouse monocyte cell line, RAW264.7. JEV infects monocytes and induces an anti-inflammatory environment by inhibiting histone deacetyl transferase. In macrophages, JEVI was found to modulate the suppressors of cytokine signaling (SOCS) to mute the pro-inflammatory nature of the host’s immune response [292]. Another reason for immune evasion might be the overexpression of indoleamine 2,3-dioxygenase (IDO) in antigen-presenting cells, followed by reduced antigen presentation [293].

JEV, similar to any other virus, modulates the cellular repertoire of microRNAs (miRNA) to invade and successfully establish its pathogenesis in the host. The role of miR-22 in JEV infection and IFN downregulation has been discussed previously. Another microRNA-432 was found to be downregulated in JEVI human brain microglial cells (CHME3). This miR-432 is associated with the phosphorylation of STAT-1 and the immune-mediated inflammatory response during viral infection. However, the downregulation is involved with SOCS upregulation, which eventually silences the host defense. Likewise, the upregulation of miR-155 during JEVI suppresses proinflammatory responses via the miRNA-based targeting of Pellino protein 1 (PELI1) [294]. These silenced PELI1 were originally responsible for inducing a pro-inflammatory response by acting as a positive regulator of MYD88 [295]. Similarly, miR-301a was found to target SOCS5 and IRF1 and repress it at the translational level [296]. Short-fragment ncRNA (sfRNA) are hallmarks of flavivirus infection and are known for blocking IFN responses, thus favoring viral pathogenesis [297] (Table 2).

#### Immune Evasion Strategies of JEV Strains

JEV differs in their geographical distribution and virulence among the five genotypes. The immune response to these genotypes is generally consistent. The immune system combats JEV through both innate and adaptive immune responses, as previously reported elsewhere [298]. Regardless of the genotype, the innate immune system is the first line of defense against JEV. It deploys interferons, cytokines, and other signaling molecules to limit the spread of the virus. Pattern recognition receptors such as TLRs, RIG-I, and MDA5 recognize viral RNA from different genotypes and activate antiviral responses accordingly [299,300].

The adaptive immune response is also consistent across JEV genotypes. B cells produce antibodies, including neutralizing antibodies that target the viral E protein, which is conserved among the genotypes. T cells, particularly CD8^+^ cytotoxic T cells, recognize and destroy virus-infected cells. CD4^+^ helper T cells support the overall immune response by producing cytokines [175]. Based on the live-attenuated SA14-14-2 strain (genotype III), the Japanese encephalitis vaccine is effective against all JEV genotypes, suggesting that the immune response is consistent across genotypes [301].

It is important to note that individual factors, such as age, immune system function, and underlying health conditions, can influence the severity of the disease and the effectiveness of the immune response. These factors might have a more significant impact on the outcome of the infection than the specific genotype of the virus [302]. The immune system combats the virus through both innate and adaptive immune responses, which effectively limit the virus’s spread and clear the infection, regardless of the specific genotype [303].

### 3.9. West Nile Viruses and Associated Immune Evasion Strategies

WNV is a neurotropic virus that, upon successful infection to the human host, activates various antiviral mechanisms in the host, including the production of IFN and pro-inflammatory cytokines followed by the expression of the anti-viral genes [304]. WNV also developed various mechanisms that facilitate the process of immune evasion. RLR signaling induces type 1 IFN production by recognizing the virus particle through PAMPs. After WNV infection, there is an inconsistency between the viral protein accumulation and IRF3 activation, indicating a possible hijacking through which WNV evades the RLRs detection [305]. Another possible mechanism is masking viral RNA, thereby allowing WNV to establish and synthesize new virus particles that eventually prevent various signaling pathways, including IFN-1 and TLR3 [306]. The viral infection also hinders IFN type I by preventing the phosphorylation of tyrosine kinase 2 (TYK2) and inhibiting the activation of STAT1 and STAT2 [307]. Furthermore, the non-structural proteins of WNV also degrade the interferon-alpha/beta receptor complex (IFNAR1) through proteasome- and lysosome-dependent pathways. However, whether this IFNAR1 degradation and phosphorylation inhibition are directly linked or act as separate events is unclear. Thus, this needs more attention to resolve the mechanism through which WNV inhibits IFN-I signaling [308]. The viral proteins, including both the structural and non-structural, that alter the IFN-I signaling process have not been identified clearly. WNV infection evades the anti-viral properties of interferon-induced protein with tetratricopeptide repeats 1 (IFIT1), an interferon-stimulated gene (ISG) protein that is highly activated after the viral infection. The virus uses the 2′-O-methyltransferase activity to escape the effects of IFIT1 as in normal scenarios; it interacts with the viral genetic material lacking the 2′-O-methylation and containing the 5′ triphosphate, thereby eventually blocking the functions of the viral proteins [309,310].

In addition to these above evading mechanisms, WNV uses other strategies, including the complement activation’s escape mechanism. The non-structural protein 1 (NS1) prevents the activation of the alternate pathway through the complement regulator factor H and facilitates the inactivation of C3b by recruiting factor I [311]. The C3b degradation leads to a less-than-optimal production of membrane attack complexes on the cell surface. The NS1 protein also hinders the activation of lectin and classical pathways by targeting the C4b-binding protein (C4BP) [312]. The higher concentration of NS1 in the extracellular space and lower levels of complement concentration eventually facilitate the inactivation of the complement system. Thus, in this manner, the viral non-structural proteins ease the evasion process from the host IFN system and the complement-dependent activation processes, thereby successfully causing the viral infection.

#### Immune Evasion Strategies by WNV Strains

The immune response to the West Nile virus can vary depending on the lineage and the individual’s immune system. However, there is limited information on the differences in immune invasion between Lineage 1 and Lineage 2 strains of WNV [313]. Some studies suggest that Lineage 2 strains may be less virulent than Lineage 1 strains, resulting in less severe disease in humans. However, more research is needed to confirm these findings and better understand the differences in pathogenicity between the two lineages [314]. In general, the immune system plays a critical role in controlling and clearing West Nile virus infection. Both innate and adaptive immune responses are involved in combating the virus. The innate immune response, which includes interferons, cytokines, and other signaling molecules, is the first line of defense against the virus [315]. The adaptive immune response, which includes T cells and antibodies, is essential for clearing the virus and providing long-term protection [316]. The differences in immune invasion between Lineage 1 and Lineage 2 strains of WNV are poorly understood. However, individual immune system strength plays a crucial role in determining the severity of the disease, regardless of the lineage.

**Table 2:** Mosquito-borne diseases and their link with post-infection other metabolic co-morbidities.

Mosquito-Borne Diseases	Year	Location	Type of Study	Method of Assessment	Disease Manifestation	Reference
Chikungunya	2005–2006	Réunion	Prospective study	Fulfilled International Encephalitis Consortium criteria for encephalitis	Perinatal encephalopathy	[317]
Chikungunya	2005–2006	Mayotte	Prospective study	Unknown	Perinatal encephalopathy	[318]
Chikungunya	2005–2006	Réunion	Prospective observational study	Seizures; EEG consistent with encephalitis	Perinatal encephalopathy	[319]
Chikungunya	2010	India	Prospective study	Altered sensorium, apnoeic seizures	Perinatal encephalopathy	[320]
Chikungunya	2014–2015	Colombia	Case series study	EEG	Perinatal encephalopathy	[321]
Chikungunya	2014–2015	Colombia	Prospective study	EEG	Perinatal encephalopathy	[322]
Chikungunya	2015	Brazil	Case study	Seizures; abnormal brain MRI	Perinatal encephalopathy	[323]
Chikungunya	2015	Honduras	Retrospective study	Unknown	Perinatal encephalopathy	[324]
Chikungunya	2016	India	Case study	Dizygotic twins; both had seizures, required ventilation, thrombocytopenia; abnormal brain MRI	Perinatal encephalopathy	[325]
Chikungunya	2016	Brazil	Case study	Prostration, lethargy, seizures, required ventilation, thrombocytopenia; abnormal brain MRI and EEG	Perinatal encephalopathy	[326]
Chikungunya	2005–2006	Réunion	Retrospective study	DIC, transient scattered parenchymal petechiae, cerebellar hematoma	Perinatal brain hemorrhage	[327]
Chikungunya	2005–2006	Réunion	Retrospective descriptive study	Unknown	Perinatal brain hemorrhage	[328]
Chikungunya	2015	Brazil	Case study	Intraventricular bleeding (cranial US), lethargy	Perinatal brain hemorrhage	[329]
Chikungunya	2005–2006	Réunion	Case study	Seizures; hypotonia	Perinatal other	[328]
Chikungunya virus	2008	France	Case study	ECG and cardiac MRI	Myopericarditis, pericardial effusion	[330]
CHIKV infection	2015	Honduras	Retrospective study	CSF analysis, EEG, CAT (brain) scan, MRI (brain)	Meningoencephalitis, seizures. CSF analysis shows enhanced leukocyte presence.	[324]
CHIKV infection	Not specified	India	Case study	Neurologic examination, sensory examination, electrophysiological studies	Global areflexia and quadriparesis. Guillain–Barre syndrome	[331]
CHIKV infection	2020	India	Case study	MRI, nerve conduction studies	Sensorimotor axonopathy, myelopathy.	[332]
Dengue	1987 to 1998	Thailand	Observational study	MRI and autopsy	Seizure, mental confusion, nuchal rigidity, spasticity of limbs, positive clonus, hyponatremia, abnormal liver enzymes and CSF pleocytosis, hemiplegia and positive Kernig	[333]
Dengue	2017	Brazil	Case study	Brain MRI	Loss of vision, generalized myalgia, severe headache, retro-ocular pain, and cutaneous rash	[206]
Dengue	1997–1999	Vietnam	Case–control study	MRI and ultrasound	Cerebral edema, seizures, hemiplegia, hemorrhage, hyponatremia, hepatic failure, and microcapillary	[205]
Dengue	2019	India	Case study	MRI (brain), cerebrospinal fluid (CSF) examination	Bradyphonia, unclear speech, gait instability, cogwheel rigidity, mask-like facies and stooped posture while walking	[204]
Dengue	2017	Sri Lanka	Case study	Electroencephalogram and cerebrospinal fluid (CSF) examination	Encephalitis	[334]
Dengue	2019	India	Prospective study	Echocardiogram, ECG	Asymptomatic sinus bradycardia, symptomatic bradyarrhythmias, left ventricular systolic dysfunction (ejection fraction 35–45%), pericardial effusion, atrial fibrillation	[335]
Dengue	1998	India	Cohort study	Echocardiogram, ECG	Ejection fraction, global hypokinesia, ST and T changes in the ECG	[336]
Dengue	1998	India	Observational study	Echocardiogram	Sinus bradycardia	[337]
Dengue	2007	Sri Lanka	Cohort study	Echocardiogram, ECG	T inversion, ST depression and bundle branch blocks, hypotension and tachycardia and bradycardia, suggestive of significant cardiac dysfunction	[338]
Dengue	2012	Brazil	Case series study	Echocardiogram, ECG	Ileo-femoral deep vein thrombosis, pulmonary thromboembolism, mesenteric vein thrombosis	[339]
Dengue	2014	México	Case report	Echocardiogram, ECG	Myocarditis characterised by: S3 gallop rhythm, generalized lung rales and shock; ECG showed sinus tachycardia, ST depression in V1-V3, and ST elevation in a VR and aVL	[340]
Dengue	2018	Sri Lanka	Case series	CT angiography, ECG	Tachycardia in three cases. Myocarditis confirmed by troponin estimation and echocardiogram in one case, and in the other two, this was also confirmed by histopathology	[341]
Dengue	2013	Singapore	Case reports	Echocardiogram, ECG	Myocarditis	[342]
Dengue	2015	Vietnam	Case report	Echocardiogram, ECG	Acute cardiac failure	[343]
Dengue	2008	India	Case report	Echocardiogram, ECG	Myocarditis, Takotsubo cardiomyopathy, ECG showed sinus bradycardia	[344]
Dengue	2016	Taiwan	Case report	Echocardiogram, cardiac biomarkers	Elevated cardiac enzymes	[345]
Dengue	2015	Singapore	Case report	Echocardiogram, ECG	Acute myocardial infarction, elevated serum troponin I levels	[346]
Dengue	2011	France	Case report	Echocardiogram, ECG	Acute pericarditis, Consulting with acute chest pain; ECG revealed negative anterolateral T waves with long QT segment	[347]
Dengue	2011	Thailand	Prospective study	Holtrer	Sinus pause, first-degree and Mobitz type I second-degree atrioventricular block (Wenckebach) and atrial and ventricular ectopic beats	[196]
Dengue	2016	India	Case report	CT angiography, ECG	Repeated symptomatic episodes of a high-degree atrioventricular block with ventricular asystole	[348]
Dengue	2015	Sri Lanka	Case report	Echocardiogram, ECG	Bradycardia and heart block with atrioventricular dissociation	[349]
Dengue	2013	India	Case series	Echocardiography	Sinus bradycardia, echocardiography showed a decreased ejection fraction	[350]
Dengue	2000	Thailand	Case reports	CT angiography, ECG	Morbitz type I second-degree atrioventricular block	[351]
Dengue	2004	Thailand	Case report	Echocardiography and ECG	Myocarditis with bradycardia	[351]
Dengue	2010	India	Case report	Echocardiography and ECG	Sino-atrial block and atrioventricular dissociation, atrial fibrillation, atrial fibrillation	[352]
Dengue	2009	Malaysia	Case report	Echocardiography and ECG	Sino-atrial block and atrioventricular dissociation, atrial fibrillation	[353]
Dengue	2003	Brazil	Case report	Echocardiography and ECG	Sino-atrial block and atrioventricular dissociation, atrial fibrillation	[354]
Dengue	2008	Saudi Arabia	Cohort study	Not specified	Acute kidney injury	[355]
Dengue	2008	Taiwan	Retrospective study	eGFR estimation	Acute kidney injury	[356]
Dengue	2009	Taiwan	Retrospective study	SCr > 2 mg/dL	Acute kidney injury	[357]
Dengue	2010	Thailand	Retrospective study	SCr > 2 mg/dL	Acute kidney injury	[358]
Dengue	2011	India	Prospective study	RIFLE	Acute kidney injury	[359]
Dengue	2012	Pakistan	Case series study	AKIN	Acute kidney injury	[360]
Dengue	2012	Taiwan	Retrospective case–control study	SCr increase ≥ 0.5 mg/dL	Acute kidney injury	[361]
Dengue	2012	India	Prospective study	AKIN	Acute kidney injury	[362]
Dengue	2015	Taiwan	Retrospective study	AKIN	Acute kidney injury	[363]
Dengue	2015	Malaysia	Retrospective study	AKIN	Acute kidney injury	[364]
Dengue	2016	India	Cohort study	KDIGO	Acute kidney injury	[208]
Dengue	2017	Taiwan	Retrospective case–control study	SCr > 2 mg/dL	Acute kidney injury	[365]
Dengue	2017	India	Observational study	SCr > 1.2 mg/dL	Acute kidney injury	[366]
Dengue	2018	India	Retrospective study	eGFR < 60 mL/min/1.73 m2	Acute kidney injury	[367]
Dengue	2018	Taiwan	Retrospective study	KDIGO	Acute kidney injury	[368]
Dengue	2018	Malaysia	Follow-up prospective cohort	AKIN	Acute kidney injury	[369]
Dengue	2018	India	Observational prospective study	AKIN	Acute kidney injury	[370]
Dengue	2019	Thailand	Retrospective single-center study	KDIGO	Acute kidney injury	[371]
Dengue	2019	India	Retrospective single-center study	Not specified	Acute kidney injury	[372]
Dengue	2019	India	Retrospective study	AKIN	Acute kidney injury	[373]
Japanese encephalitis	2014	China	Case study	EEG, MRI, electromyography, and CSF analysis	Guillain–Barre syndrome	[374]
Japanese encephalitis	2012	India	Case study	MRI (cervico-thoracic spine)	Acute transverse myelitis	[375]
Malaria	2014	India	Observational study	Neurological and neuropsychiatric evaluation by MSME (Mini-Mental Status Examination) and BRPS (Brief Psychiatric Rating Scale)	Cerebellar ataxia, psychosis, convolution	[376]
Malaria	2011	Sierra Leone	Case study	MRI and autoimmune screen	Extensive abnormal high signal and swelling in brainstem and spinal cord	[377]
Malaria	1965–1994, 2010–2015	Ghana and Uganda	Case–control study	Multiplex Luminex bead assay, histological and cytological investigations	Burkitt Lymphoma	[378]
Malaria	2005–2010	Malawi	Case–control study	Histological and cytological laboratory investigations	Burkitt Lymphoma	[379]
Malaria	2011–2015	Uganda	Case–control study	Histological and cytological laboratory investigations	Burkitt Lymphoma	[380]
Malaria	2010–2016	Uganda, Tanzania, and Kenya	Case–control study	Histological and cytological laboratory investigations	Burkitt Lymphoma	[380]
Malaria	1987–2015	Sweden	Cohort study	Histological and cytological laboratory investigations	Burkitt Lymphoma	[381]
Malaria	2017	South Korea	Case Study	Brain MRI	Parkinsonian features	[382]
Malaria	2015	India	Case study	CT scan of the brain, MRI, and electroencephalography	Cognitive dysfunction, calcification (lesion) in the tempo-parietal region, temporal and hippocampal hyperintensities, microhaemorrhages	[383]
Malaria	1999	India	Case study	Neurological (nerve conduction) examination	Guillain–Barre syndrome	[384]
West Nile Virus	2022	USA	Case study	ECG, transthoracic echocardiogram (TTE), X-ray study	Reduced LV systolic	[385]
West Nile Virus	2022	USA	Case study	TTE and X-ray	Left ventricular hypokinesis	[386]
West Nile virus (WNV)	2016	USA	Prospective cohort study	Biomicroscopy	Retinopathy and Neurological and Neurocognitive Sequelae	[387]
West Nile virus (WNV)	2002–2004	USA	Prospective cohort study	ELISA and anti-WNV IgM antibody detection	Encephalitis/meningoencephalitis/encephalomyelitis (WNE)	[388]
Yellow fever virus	2016	Portugal	Case study	CT scan; CSF investigation	Acute onset expression aphasia, agraphia, dyscalculia	[389]
Yellow fever virus	2020	Brazil	Experimental study	Histological and cytological laboratory investigations	Hepatic injury	[390]
ZIKV infection	2016	France	Case study	MRI (Spinal), electromyography, and CSF examination	Acute myelitis and lesions of the cervical and thoracic spinal cord	[391]
ZIKV infection	2017	Brazil	Case study	Brain MRI, electroencephalography, and autopsy	Meningoencephalitis.	[392]
ZIKV infection	2015	Colombia	Observational study	Electromyography and nerve conduction studies, CSF examination	Guillain–Barre syndrome (inflammatory demyelinating polyneuropathy)	[393]
ZIKV infection	2017	Colombia	Case–control study	MRI and electrophysiological examination	Encephalitis, peripheral facial palsy, thoracolumbosacral myelopathy, and transverse myelitis	[394]
ZIKV infection	2017	Brazil	Cross-sectional study	MRI (brain) and EEG examination	Epilepsy tended to be early and refractory	[395]

## 4. Immunological Aspects Facilitating Therapeutic Development

Developing an effective vaccine against malaria has been challenging, as the parasite has evolved various mechanisms to evade the host’s immune response. However, recent studies have provided new insights into the immune mechanisms involved in malaria and potential targets for vaccine development [396]. However, the high genetic diversity of *Plasmodium* parasites poses a challenge to developing a vaccine that targets a single antigen. Therefore, researchers are exploring the use of multi-antigen vaccines or vaccines that target conserved regions of the parasite’s genome [397]. Another approach for vaccine development is to target the host’s immune response. Studies have shown that the immune response to malaria is complex and involves various components of both the innate and adaptive immune systems. For example, the production of type 1 interferons by dendritic cells has been shown to be important in controlling the parasite in animal models. Targeting these immune response components may lead to the development of more effective vaccines [398]. Additionally, recent studies have focused on understanding the role of the gut microbiome in modulating the host’s immune response to malaria. The gut microbiome has been shown to play a critical role in shaping the host’s immune system, and perturbations in the gut microbiome have been associated with increased susceptibility to malaria. Therefore, targeting the gut microbiome may also provide a novel avenue for vaccine development [399]. New insights into the immune mechanisms involved in malaria and the role of the gut microbiome in modulating the host’s immune response have provided potential targets for vaccine development. Recently, a randomized clinical trial was conducted for “R21 in adjuvant Matrix-M” in Burkina Faso. Its efficacy surpasses the previously most effective vaccine candidate, RTS,S/AS01 [400]. One major immune mechanism involved in malaria is the production of antibodies that target specific parasitic antigens [401].

The potential defenses against flavivirus infection, including DENVs, were thought to depend on whether the NS1 inductive antibodies had a complement-fixing function. The NS1 protein of DENV has many unique properties that make it special for vaccine development. Early studies suggested that the purified NS1 protein could serve as a subunit vaccine candidate for a variety of flaviviruses. Another target against dengue could be creating a recombinant subunit vaccine utilizing the E protein, which may result in a long-lasting protective immune response [402].

The glycoproteins E2 and E1, which are found on the envelope of CHIKV, facilitate the development of subunit vaccines [403]. Animal studies on YFV showed that YFV NS1 inductive antibodies with complement-fixing (CF) activity showed a protective function, while those without CF activity did not, pointing to the crucial role that vaccine-induced CF antibodies play in protection [404].

Vaccine development strategies for the Zika virus include mRNA vaccines, DNA vaccines, and virus-like particle vaccines. DNA vaccines are generally encoded from the viral coding sequence of prM-E regions [405]. As vaccine candidates for various infectious diseases, in vitro transcribed mRNAs that substitute 1-methyl pseudouridine for uridine or 5-methylcytidine for cytidine have now been used [406]. By using this strategy, various RNA vaccines have been developed against ZENV [407].

Furthermore, the DNA vaccine strategy for developing vaccines against RVFV includes encoding of the M segment at the second or fourth methionine that codes for Gn and Gc with (RVFV + NSm DNA) or without (RVFV-NSm DNA) the Nsm protein, respectively [408].

## 5. Conclusions

Mosquito-borne diseases are major issues in tropical countries. These diseases are caused by various pathogens, such as viruses (DENV, CHIKV, ZIKV, WNV, JEV, YFV, and RVFV) and protozoa (*Plasmodium*). The initial immune response against mosquito-borne infections is nearly the same across various species of parasites and viruses. In contrast, certain strains at different stages of infection are found to alter the immune microenvironment according to their pathological needs. The infection stimulates the host’s immune system, leading to other metabolic co-morbidities, such as cardiovascular, neurological, neurodegenerative, and renal disorders and carcinoma. This highly complex process involves both the adaptive and innate immune systems, which exert their effects through various molecular and regulatory pathways through changes in the basic nature of cells. The intricate lifecycle and ever-changing antigens present in malaria pose significant challenges to creating an effective vaccine. To improve the potency of treatment, adopting a dual approach that bolsters T cell-mediated immunity during the liver stages and enhances antibody-driven immunity in the blood stages may be beneficial. In the context of mosquito-borne viral diseases, strategies often focus on addressing viral structural and non-structural proteins. Through this review, we have discussed the immune metabolic pathways of each disease in depth so that in the future, we can deepen our understanding of the suitability of each disease for vaccine discovery. We can also increase our understanding of finding new avenues for suitable drug targets for mosquito-borne diseases.

## Figures and Tables

**Table 1:** Different *Plasmodium* sp. and its immune responses.

*Plasmodium* Species	Severity	Distribution	Innate Immunity	Humoral Immunity	Major Involved Pathway	References
*P. falciparum*	High	Africa, Asia, and Latin America	Neutrophils, monocytes, and NK cells	Long-lived plasma B cells and memory B cells; antibodies	Toll, immune deficiency (Imd), Janus kinase (JNK), and signal transducers and activators of transcription (STAT)	[52,53]
*P. vivax*	Moderate	Asia, Latin America, Africa	Cytokines, dendritic cells	B cells and antibodies	IFN-α/IFN-γ and TCR pathway, MAPK6/MAPK4 signalling	[46,54]
*P. ovale*	Low–moderate	Africa, Asia	NA	NA	NA	
*P. malariae*	Low	Africa, Asia, and Latin America	NA	NA	NA	
*P. knowlesi*	Moderate–high	Southeast Asia	NA	NA	NA	

(NA = Not available).

## Data Availability

The data presented in this study are available in the manuscript.
